# Preexisting helminth challenge exacerbates infection and reactivation of gammaherpesvirus in tissue resident macrophages

**DOI:** 10.1371/journal.ppat.1011691

**Published:** 2023-10-17

**Authors:** Christina M. Zarek, Chaitanya Dende, Jaime Coronado, Mihir Pendse, Phillip Dryden, Lora V. Hooper, Tiffany A. Reese

**Affiliations:** 1 Department of Immunology, University of Texas Southwestern Medical Center, Dallas, Texas, United States of America; 2 Department of Microbiology, University of Texas Southwestern Medical Center, Dallas, Texas, United States of America; 3 The Howard Hughes Medical Institute, University of Texas Southwestern Medical Center, Dallas, Texas, United States of America; The University of North Carolina at Chapel Hill School of Medicine, UNITED STATES

## Abstract

Even though gammaherpesvirus and parasitic infections are endemic in parts of the world, there is a lack of understanding about the outcome of coinfection. In humans, coinfections usually occur sequentially, with fluctuating order and timing in different hosts. However, experimental studies in mice generally do not address the variables of order and timing of coinfections. We sought to examine the variable of coinfection order in a system of gammaherpesvirus-helminth coinfection. Our previous work demonstrated that infection with the intestinal parasite, *Heligmosomoides polygyrus*, induced transient reactivation from latency of murine gammaherpesvirus-68 (MHV68). In this report, we reverse the order of coinfection, infecting with *H*. *polygyrus* first, followed by MHV68, and examined the effects of preexisting parasite infection on MHV68 acute and latent infection. We found that preexisting parasite infection increased the propensity of MHV68 to reactivate from latency. However, when we examined the mechanism for reactivation, we found that preexisting parasite infection increased the ability of MHV68 to reactivate in a vitamin A dependent manner, a distinct mechanism to what we found previously with parasite-induced reactivation after latency establishment. We determined that *H*. *polygyrus* infection increased both acute and latent MHV68 infection in a population of tissue resident macrophages, called large peritoneal macrophages. We demonstrate that this population of macrophages and vitamin A are required for increased acute and latent infection during parasite coinfection.

## Author summary

Coinfections, or concomitant infections with different pathogens, happen regularly in the natural world. We investigated coinfection with an intestinal parasite and a gammaherpesvirus in mice to determine how prior helminth challenge affects chronic herpesvirus infection and reactivation. In our previous reports where we infected with gammaherpesvirus first followed by intestinal parasite, we found that parasite challenge increased reactivation of the virus. In this study, we find that prior infection with helminth leads to increased viral infection of tissue resident macrophages during acute and chronic herpesvirus infection. During the chronic phase of infection, the coinfected mice had more latent infection in tissue resident macrophages and reactivated more herpesvirus. This work suggests that helminth infections exacerbate chronic herpesvirus infection. Importantly, the mechanism by which this occurs is dependent on the order of coinfection.

## Introduction

Research on pathogenesis and the immune response to infections, particularly in animal models, focuses on single infectious challenges. In reality, hosts are exposed to multiple pathogens over the course of their life, either simultaneously or sequentially. Although the research on coinfections is increasing, we have only observed the tip of the iceberg. In designing coinfection experiments, researchers often decide on a specific order and timing of coinfection and rarely test whether the outcome of coinfection is different when the sequence of infections is altered. It is important to know how order of infection changes the outcomes of coinfection because humans likely experience all possibilities of infection sequences. In a system we developed in mice with gammaherpesvirus and intestinal parasite coinfection we asked whether the sequence of coinfection led to different outcomes.

Murine gammaherpesvirus-68 (MHV68) is the mouse model for the human gammaherpesviruses, Epstein-Barr virus and Kaposi’s sarcoma associated herpesvirus (KSHV). Similar to all herpesviruses, MHV68 establishes a chronic latent infection characterized by limited to no viral replication and gene expression [1,2]. Gammaherpesviruses establish latency in B cells, macrophages, and dendritic cells [3], and the maintenance of latency is a tightly controlled process leading to the same frequency of latently infected cells regardless of the infectious dose [4]. Reactivation of latent virus is induced by multiple signals, including but not limited to HDAC inhibitors in macrophages [5] and B cell activation [6]. Previous work by us demonstrated that coinfection with a helminth, *Heligmosomoides polygyrus*, during latency of MHV68 triggered *in vivo* reactivation in a STAT6 dependent mechanism [7].

*Heligmosomoides polygyrus* (HP) is a chronic mouse helminth that is a model for human hookworm infection [8]. In the mouse, HP resides exclusively in the gastrointestinal tract, primarily the duodenum [9]. Despite having an entirely gastrointestinal lifestyle, HP has multiple immunomodulatory mechanisms that can impact the systemic immune response [10–12]. Such systemic effects help demonstrate the importance of studying helminths, especially in the context of coinfection. One consequence of HP infection is the expansion of large peritoneal macrophages (LPMs) [13,14]. LPM expansion is independently driven by IL-4 and CSF-1 during HP infection [13], but it is unknown if the expansion plays a role in immunity to HP.

Despite how common gammaherpesvirus and helminth infections are in certain parts of the world, coinfections with chronic pathogens are understudied. Coinfections between human gammaherpesviruses and helminths are common in areas where herpesvirus-driven cancers are endemic, especially in Sub-Saharan Africa [15]. Moreover, a study showed that seroprevalence against KSHV increased with increasing hookworm burden in Ugandan women [16]. Given the complexity of these associations in humans, mouse models of coinfections are valuable tools to untangle the interactions of these pathogens with the immune system.

We previously determined that HP infection during latent MHV68 infection induced transient viral reactivation from latency. We demonstrated that this transient viral reactivation required IL-4/IL-13 signaling via the transcription factor, STAT6, which is bound to the latent-to-lytic switch gene promoter of ORF50/RTA [7]. We further determined that IL-4 signaling specifically induced viral reactivation in macrophages, but not in B cells [17]. All these experiments examined the effects of parasite and IL-4 stimulation on viral reactivation after latency was already established. However, whether HP infection alters latency and reactivation if the parasite infection occurs first, before MHV68 infection, has not been examined.

We sought to determine whether the order of HP and MHV68 challenge led to the same phenotype. To examine whether prior infection with HP altered MHV68 latency and reactivation, we chose a coinfection model where we infected with HP first, followed by MHV68 seven days later. We found that prior HP infection increased MHV68 reactivation from latency, but in a manner independent of STAT6, indicating that the order of coinfection leads to mechanistically distinct phenotypes.

## Results

### Prior infection with *H*. *polygyrus* increases MHV68 reactivation

**Fig 1:**
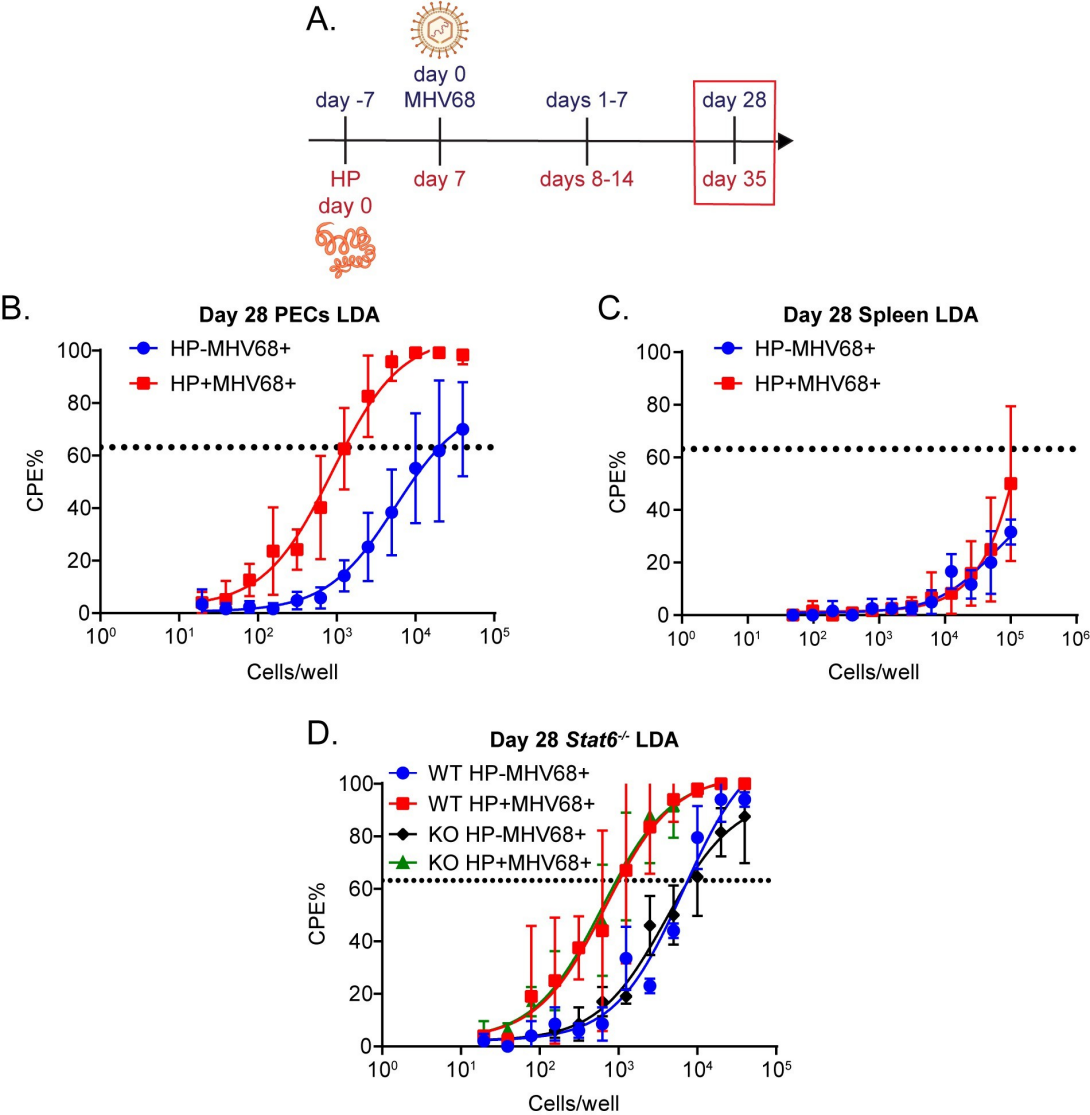
Coinfection with *H*. *polygyrus* increases *ex vivo* reactivation independently of STAT6. (A) HP was infected by oral gavage 1 week prior to MHV68 infection. HP-infected or uninfected mice were challenged with 10^6^ PFU of MHV68 intraperitoneally. PECs and spleen were isolated at day 28–31 of MHV68 infection. Approximate timepoint outlined by red box. Partially created using BioRender. (B) Limiting dilution assays were performed from C57BL/6 PECs. Data pooled from 4 independent experiments (3 mice pooled/group). (C) Limiting dilution assays were performed from C57BL/6 splenocytes. Data pooled from 4 independent experiments (3 mice pooled/group). (D) *Stat6*^*-/-*^ or littermate control mice were infected and LDAs were performed at day 28–29 of MHV68 infection. Data are pooled from 2 independent experiments (3 mice pooled/group). Dotted line represents Poisson distribution.

Our first question was whether mice with prior parasite infections would have increased viral reactivation. Mice were infected with HP or left uninfected and seven days later challenged with MHV68 intraperitoneally (Fig 1A). Our previous studies infected with MHV68 intraperitoneally and found reactivation specifically in peritoneal macrophages following HP infection or intraperitoneal IL-4 treatment; therefore, we maintained the dose and route of infection [7,17]. Twenty-eight days after MHV68 challenge, which is a timepoint when latency is established, we explanted live peritoneal exudate cells (PECs) and splenocytes for a limiting dilution assay (LDA) to measure spontaneous virus reactivation [18]. PECs and splenocytes were plated in serial two-fold dilutions on mouse embryonic fibroblasts and cytopathic effect (CPE) was quantified three weeks later. In PECs, macrophages are the primary cell type that reactivates MHV68, while in the spleen, reactivation primarily occurs in B cells [19–21]. We found that HP/MHV68 coinfected mice had a 16-fold higher reactivation frequency in PECs than mice that received only virus (1 out of 1,267 cells versus 1 out of 20,231 cells, respectively) (Fig 1B). In spleen, the frequency of reactivation was below Poisson’s distribution, and was not significantly different between HP/MHV68 coinfected mice and virus only mice (Fig 1C). These results suggest that coinfection with HP increases reactivation from macrophages in the peritoneal cavity, but not from B cells in the spleen (Fig 1B and 1C). We also performed LDA’s with disrupted samples of PECs and splenocytes to quantify any preformed virus that replicated *in vivo*. Disrupted samples are prepared from aliquots of the samples used for the regular LDA’s and are mechanically lysed to free any preformed virus. Two-fold dilutions are plated on the MEFs, as before. There was no preformed virus from either group or tissue, suggesting that the virus was latent *in vivo* prior to explant (S1A and S1B Fig). This data suggests that prior HP infection increases the propensity of the peritoneal cells to reactivate. Notably, this is distinct from our previous study where we found that HP challenge during latency induced virus reactivation transiently *in vivo*, as measured by increased bioluminescence signal from a marker virus [7]. Further, since we saw no phenotype for splenic reactivation, we chose to focus on the effect of HP coinfection on PECs.

To determine whether MHV68 infection altered the establishment of chronic HP infection, we examined worm burden and fecundity between HP-only infected mice and HP/MHV68 coinfected mice. There was no significant difference in worm burden or fecundity at day 35 of HP infection (S1C Fig), demonstrating that MHV68 infection does not impact the control of chronic HP infection.

Because our previous work indicated that challenge with HP during MHV68 latency increased *in vivo* reactivation in a STAT6-dependent manner [7] and peritoneal macrophages respond to IL-4 signaling leading to proliferation and activation of STAT6 dependent gene expression [13,22], we examined whether increased virus reactivation in mice with preexisting helminth infection required STAT6 signaling. We infected *Stat6*^-/-^ mice and their littermate controls and performed LDAs. We found that HP/MHV68 coinfected *Stat6*^-/-^ mice still had increased *ex vivo* reactivation compared to MHV68-infected *Stat6*^-/-^ mice (Fig 1D). The *Stat6*^-/-^ mice also did not have any preformed virus in disrupted samples (S1D Fig), demonstrating that HP infection before MHV68 does not initiate *in vivo* reactivation. This confirmed that STAT6 signaling was not driving the increased *ex vivo* reactivation in coinfected mice when HP infection occurs before MHV68 infection.

### Coinfected mice have increased infection in tissue-resident macrophages during latency

**Fig 2:**
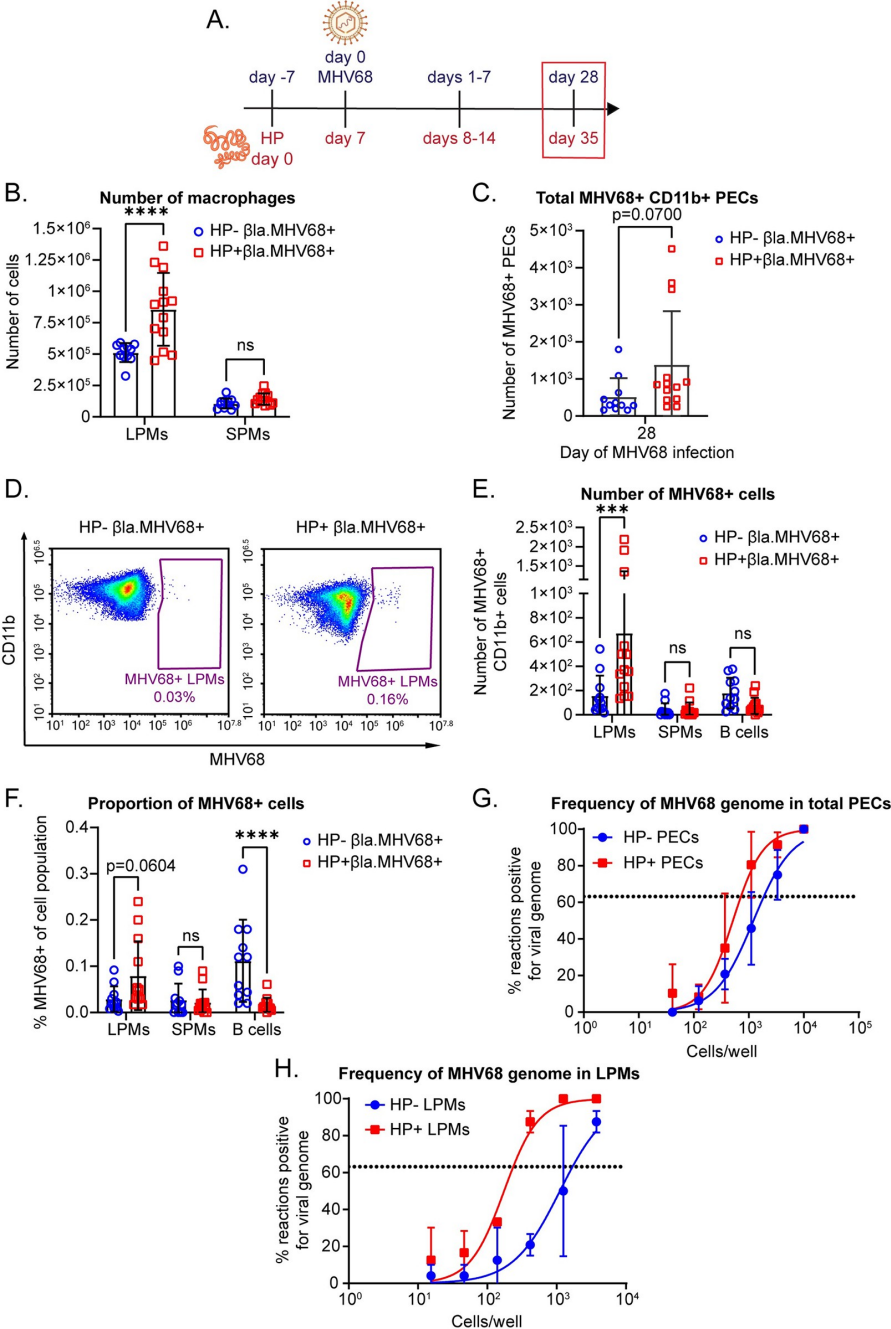
Coinfected mice have increased infection in tissue-resident macrophages during latency. (A) Timeline of infections with HP by oral gavage and MHV68 by intraperitoneal injection. The timepoints shown in (B-G) are outlined by a red box. Partially created in BioRender. (B) Quantification of flow cytometric analysis of the number of LPMs and SPMs at day 28 of MHV68 infection. Data are pooled from 3 independent experiments (n = 11-13/group, mean ± standard deviation). Each dot represents an individual mouse. (C-F) Quantification of flow cytometric analysis of MHV68-infected CD11b+ PECs at day 28 of MHV68 infection. CD11b+ cells were isolated with CD11b+ beads and MACs columns before staining. LPMs were gated as CD19- CD11b^hi^ ICAM-2^hi^. SPMs were gated as CD19- CD11b+ ICAM-2^lo^. B cells were gated as CD19+. Data are pooled from 3 independent experiments (n = 11-13/group, mean ± standard deviation). Each dot represents an individual mouse. (C) Total number of MHV68-infected CD11b+ PECs. (D) Representative flow plots of MHV68+ LPMs. (E) Quantification of number of MHV68-infected macrophages and B1 B cells. (F) Proportion of MHV68-infected cells out of the parent populations. (G) Total PECs from days 28–31 of MHV68 infection were subjected to limiting dilution PCR analysis to detect frequency of viral genomes. Data are pooled from 4 independent experiments (3 mice pooled/ group, mean± standard deviation). (H) LPMs were sorted from mice at days 28 and 29 of MHV68 infection as CD19- SiglecF- Ly6G- F4/80+ CD11b^hi^ ICAM-2^hi^. Sorted cells were subjected to limiting dilution PCR analysis to detect frequency of viral genomes. Data are pooled from 2 independent experiments (4–8 mice were pooled for each group before sorting, mean± standard deviation). (B, D, E) P-values, 2-way ANOVA, Tukey’s multiple comparisons. (C) P-values, unpaired t-test. (G, H) Dotted line represents Poisson distribution. * P ≤ 0.05, ** P ≤ 0.01, *** P ≤ 0.001, **** P ≤ 0.0001.

Because we noted increased viral reactivation in coinfected mice, we questioned if the number and type of virally infected cells was different. We collected PECs at day 28 of MHV68 infection and examined the cell composition by flow cytometry (Fig 2A). We found that HP/MHV68 coinfected mice had a higher number of large peritoneal macrophages (LPMs) than virus-only infected mice, but that there was no difference in the number of small peritoneal macrophages (SPMs) between the two groups (Fig 2B). LPMs are the tissue resident population of macrophages that derive from an embryonic precursor, while SPMs arise from monocytes that differentiate following recruitment from the blood. IL-4 and parasite infection induce expansion of LPMs [13,14,23]. In addition, one report found that LPMs were the main cell type infected by MHV68 in the peritoneal cavity when the virus was injected intraperitoneally [24].

We next examined if parasite infection increased MHV68 infection in peritoneal cells. To do this we used a reporter virus, MHV68.ORF73β-lactamase. This virus was described by Nealy, et al, and encodes the bacterial enzyme β-lactamase fused to a constitutively active viral promoter, ORF73 [24–27]. Infected cells at any stage of MHV68 infection may express β-lactamase, which allows for the detection of those infected cells by flow cytometry after incubation with a substrate for β-lactamase, CCF4-AM [24,25]. To enhance our detection of latently infected cells, we first isolated CD11b+ PECs using positive selection beads. Since the majority of CD11b+ PECs are cell types that can be infected by MHV68 (LPMs, SPMs, and B1 B cells [3,20,24]), isolating CD11b+ PECs before detection of β-lactamase enriches for MHV68+ cells (S2 and S3 Figs for gating strategies). We found that the HP/MHV68 coinfected mice had increased numbers of virus infected CD11b+ PECs compared to virus-only mice (Fig 2C), although this was not statistically significant. Next, we examined which populations of cells were infected during latency and found that there were increased infected LPMs, but not SPMs or B cells, in the HP/MHV68 coinfected mice (Fig 2D and 2E). HP/MHV68 coinfected mice had a trend of an increased proportion of infection in the LPMs, while there was no difference in the proportion of infected SPMs between groups (Fig 2F). Conversely, HP/MHV68 coinfected mice had a decreased proportion of infected B cells during latency (Fig 2F).

It is possible that MHV68.ORF73β-lactamase virus underestimates the number of MHV68 infected cells [24,25]. To validate our observations that there were increased MHV68-infected LPMs in coinfected mice, we measured the frequency of latency using a different assay: limiting-dilution PCR (LD-PCR), which is a well-established assay in the field to quantitate viral genomes and is used in parallel with the LDA reactivation assay to distinguish the presence of viral genome from actual reactivation [20]. LD-PCR measures the frequency of MHV68 viral genomes by nested PCR for a MHV68 gene, ORF72, and is sensitive down to one copy of viral genome [20]. We first analyzed total PECs, which include LPMs, SPMs, B cells, and cell types that do not harbor MHV68. When we compared the point at which the dilution curves crossed Poisson’s distribution, we observed that the frequency of viral genome in PECs from HP/MHV68 coinfected mice was approximately 1 out of 722 PECs, while the frequency of viral genome from MHV68-only infected mice was approximately 1 out of 1,882 PECs (Fig 2G). HP/MHV68 coinfected mice had approximately a 2.5-fold increase in the frequency of genome in total PECs. To specifically determine the frequency of viral genome in LPMs, we sorted LPMs from HP/MHV68 coinfected or MHV68-infected mice and then performed LD-PCR. We found that there was a 7-fold increase in the frequency of viral genome in sorted LPMs from HP/MHV68 coinfected mice (1 out of 237 LPMs) compared to MHV68-only infected mice (1 out of 1,698 LPMs) (Fig 2H). Similar to the data obtained from the MHV68.ORF73β-lactamase reporter virus, we found that total PECs have a trend towards increased latent infection, while LPMs from HP/MHV68 coinfected mice have a significant increase in the frequency of latency. This suggests that coinfection with HP either promotes a higher frequency of latency establishment or better maintenance of the latently infected cells. Overall, this data demonstrates that coinfection promotes increased latency in LPMs.

It is worth noting that the quantification of MHV68+ cells by flow cytometry in these experiments and subsequent experiments in the paper is highly variable with a significant spread in data points. To be fully transparent, we have shown individual mice as data points, rather than only bar graphs with error bars. This variability is particularly striking for the flow cytometry experiments during viral latency. We think this variability results from both the inherent irregularity that arises from collecting peritoneal washes, as well as the variability that occurs with detection of rare populations of cells, such as latently infected cells, by flow cytometry. To overcome this particular challenge, we enriched MHV68+ cells by isolating CD11b+ cells with positive selection beads before analyzing by flow cytometry. This reduces the background from cell populations that do not harbor latent MHV68. We also perform experiments multiple times to achieve high enough sample size.

### LPM expansion during intestinal parasite infection increases viral infection at acute timepoints in the peritoneal cavity

**Fig 3:**
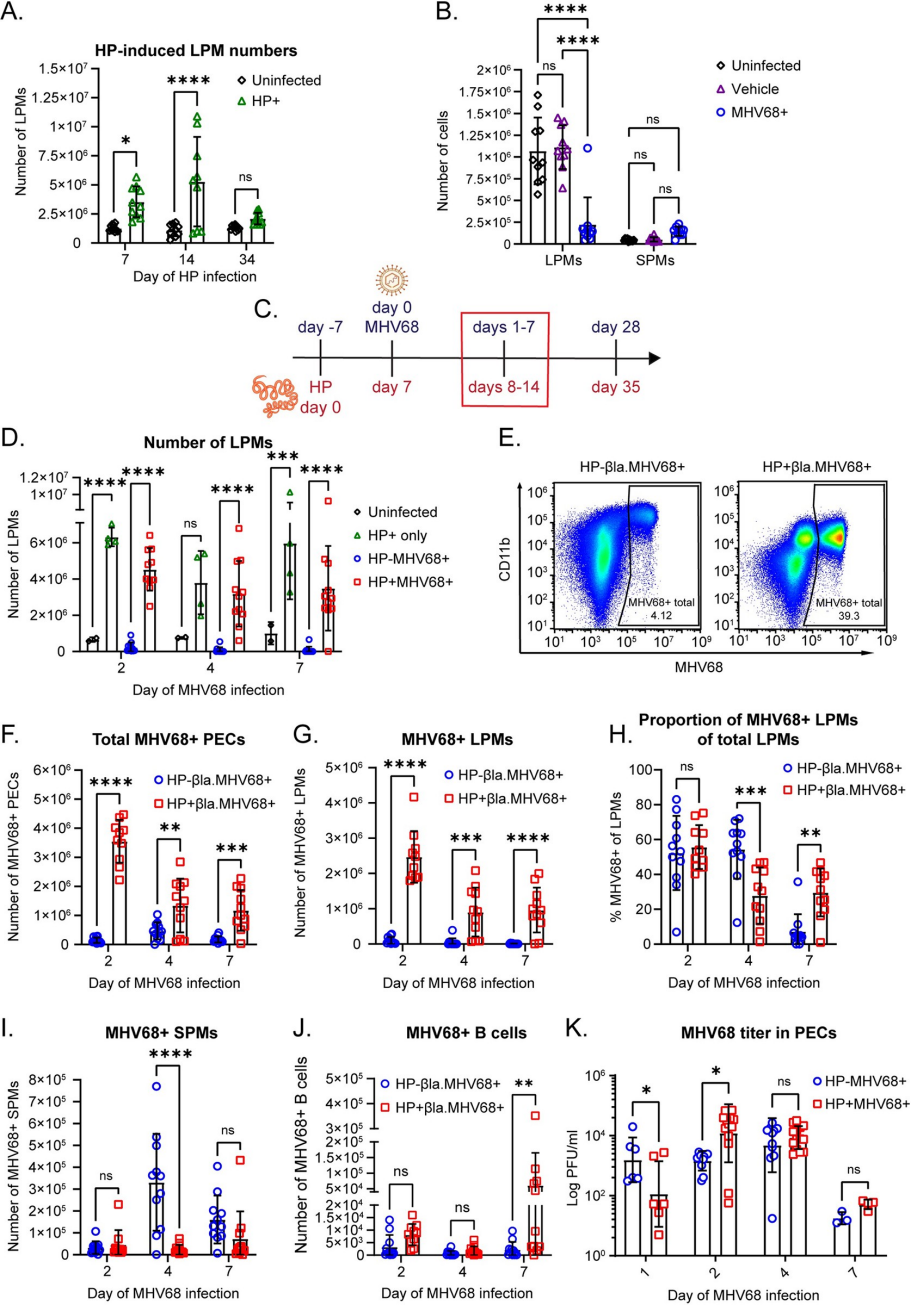
LPM expansion during intestinal parasite infection increases viral infection in the peritoneal cavity. (A) C57BL/6J mice were infected with 100 L3 larvae of HP by oral gavage. Quantification of flow cytometric analysis of LPMs in C57BL/6 mice at the indicated days post HP infection. LPMs were gated as CD3- CD19- Siglec F- CD11b^hi^ ICAM-2^hi^. Data are pooled from 3 independent experiments (n = 9-10/group, mean ± standard deviation). (B) C57BL/6J mice were left untreated and uninfected (Uninfected), intraperitoneally injected with sterile PBS (Vehicle) or intraperitoneally infected with 10^6^ PFU of MHV68 (MHV68+). PECs were collected on day 4 of MHV68 infection and the number of LPMs was quantified by flow cytometry. LPMs were gated as CD3- CD19- Siglec F- CD11b^hi^ ICAM-2^hi^. Data are pooled from 2 independent experiments (n = 10/group, mean ± standard deviation) (C) Timeline of infections with HP by oral gavage and MHV68 by intraperitoneal injection (10^6^ plaque forming units (PFU)). The time points shown in (D-K) are outlined by a red box. Partially created in BioRender. (D) Quantification of flow cytometric analysis of LPMs in C57BL/6 mice at days 2, 4, and 7 post MHV68 infection. LPMs were gated as CD19- CD11b^hi^ ICAM-2^hi^. Data are pooled from 3 independent experiments (n = 10–11 MHV68+ groups, n = 4 HP+ only group, n = 2 uninfected group, mean ± standard deviation). (E-J) Mice were infected as in (C) with 10^6^ PFU of MHV68.ORF73β-lactamase virus. Quantification of flow cytometric analysis of MHV68-infected PECs at days 2, 4, and 7 post MHV68 infection. Data are pooled from 3 independent experiments (n = 10-11/group, mean ± standard deviation). (E) Representative flow plots of the total β-lactamase-positive (MHV68+) PECs. (F) Quantification of the total number of MHV68-infected PECs. (G) Number of MHV68-infected LPMs. (H) Proportion of LPMs that are MHV68-infected. (I) Number of MHV68-infected SPMs. SPMs were gated as CD19- CD11b+ ICAM-2^lo^. (J) Number of MHV68-infected B cells. B cells were gated as CD19+. Each dot represents an individual mouse. (K) MHV68 titers determined by plaque assay of PECs at day 1–7 of infection. Data are pooled from 5 independent experiments (n = 6–7 day 1, n = 11–14 days 2 and 4, n = 3–4 day 7, mean ± standard deviation). P-values, 2-way ANOVA, Tukey’s multiple comparisons * P ≤ 0.05, ** P ≤ 0.01, *** P ≤ 0.001, **** P ≤ 0.0001.

We observed increased total LPMs and increased virally infected LPMs in HP/MHV68 coinfected mice during latency. This led us to investigate whether HP coinfection prevented the macrophage disappearance reaction previously observed during acute viral and bacterial challenges in the peritoneal cavity [24,28]. We first verified that HP infection expands LPMs in our system, as reported previously [13,14]. To do this, we compared the number of LPMs between uninfected and HP infected mice at days 7, 14, and 34 post HP infection (S4 Fig for gating strategy). We found that the number of LPMs was elevated in HP-infected mice at days 7 and 14 but had returned to homeostatic levels by day 34 (Fig 3A). This confirmed that in our facility HP infection causes expansion of LPMs. Additionally, to verify the macrophage disappearance reaction (MDR) during MHV68 infection [24], we compared the number of LPMs between uninfected mice, PBS injected mice, and MHV68 infected mice at day 4 of infection. We found that MHV68 infected mice had significantly reduced LPM numbers compared to both uninfected groups, indicative of MDR (Fig 3B).

Once we verified that we saw LPM expansion with HP infection and MDR with MHV68 acute infection, we next examined HP/MHV68 coinfection. As before, we infected mice with HP and then infected MHV68 intraperitoneally seven days later (Fig 3C). Similar to HP-only infected mice, HP/MHV68 coinfected mice had increased numbers of LPMs at days 2, 4, and 7 of MHV68 infection (Fig 3D), whereas MHV68-only infected mice had lower numbers of LPMs during the acute timepoints (Fig 3D) [24]. These data indicate that HP coinfection expanded LPMs and prevented MDR in the peritoneal cavity upon MHV68 challenge.

We next quantified the number and proportion of MHV68-infected cells at acute timepoints in single vs coinfected mice (S5 Fig for gating strategy). We quantified the total number of infected PECs and found that HP/MHV68 coinfected mice had increased numbers of MHV68-infected total PECs (Fig 3E and 3F). When we examined the number of infected LPMs during coinfection, we found that HP/MHV68 coinfected mice had increased numbers of infected LPMs at days 2, 4, and 7 of MHV68 infection (Fig 3G). Assessment of the proportion of infected LPMs revealed an equivalent percent of MHV68-positive cells at day 2, a decrease at day 4 and an increase at day 7 in HP/MHV68 coinfected mice (Fig 3H). Other cell types that are infected with MHV68 in the peritoneal cavity include SPMs and B cells [3,20,24]. We found that there was a decreased number of infected SPMs at day 4 of MHV68 infection in HP/MHV68 coinfected mice, but no difference at days 2 and 7 (Fig 3I). There was an increased number of infected B cells at day 7 of infection in HP/MHV68 coinfected mice, but no difference at days 2 or 4 (Fig 3J). Because the absolute number of infected SPMs and B cells is far fewer than infected LPMs (Fig 3G, 3I and 3J), we concluded that HP-induced expansion of LPMs led to increased MHV68 infection, particularly in these cells.

Since we observed that HP/MHV68 coinfected mice had increased infection compared to MHV68-only mice, we questioned whether that was caused by increased replication of the virus. Notably, the detection of infected cells using the β-lactamase expressing virus does not indicate that the cells are replicating MHV68. Instead, virus-positive cells could have latent MHV68. To determine if the increased infection in HP/MHV68 coinfected mice was due to differences in virus replication, we measured viral titer in the peritoneal cavity. We found that HP/MHV68 coinfected mice had decreased titer at day 1 of MHV68 infection, increased titer at day 2 of infection, and no difference in titer at days 4 and 7 of infection (Fig 3K). This data suggests that the increased infection seen in coinfected mice over multiple timepoints is not caused by increased replication of MHV68. Since there is a consistently higher number of infected LPMs at days 2, 4, and 7 of MHV68 infection, but not higher titer at all these timepoints, it is likely that MHV68 is latent in most of the infected LPMs, rather than replicating. Overall, we demonstrated that prior HP infection prevents MDR upon MHV68 challenge and increases viral infection in LPMs throughout acute and latent MHV68 infection.

### HP-independent expansion of LPMs, but not SPMs, is sufficient to cause increased MHV68 infection during the acute stage of viral infection

**Fig 4:**
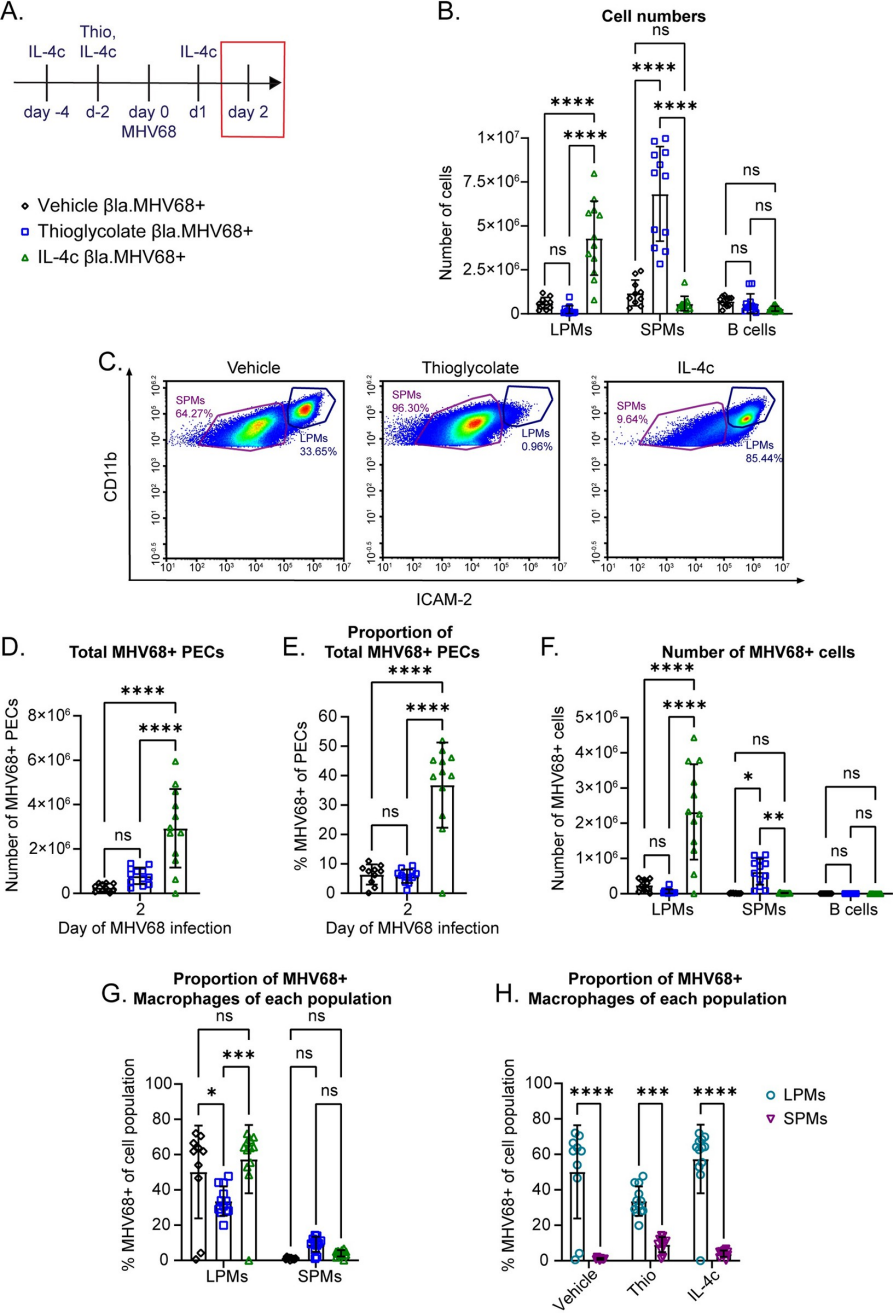
IL-4c-induced LPM expansion is sufficient to increase viral infection in the peritoneal cavity during acute MHV68 infection. (A) Timeline of intraperitoneal IL-4 complex (IL-4c), thioglycolate (Thio) treatments and MHV68.ORF73β-lactamase intraperitoneal infection (10^6^ plaque forming units (PFU)). 5μg of IL-4 and 25 μg α-IL-4 were complexed and injected for LPM expansion. 3.8% sterile thioglycolate broth was used to expand SPMs. The time point shown in (B-H) is outlined by a red box. (B) Quantification of flow cytometric analysis of LPMs, SPMs, and B cells at 2 days post MHV68 infection. LPMs were gated as CD19- CD11b^hi^ ICAM-2^hi^. SPMs were gated as CD19- CD11b+ ICAM-2^lo^. B cells were gated as CD19+. Data are pooled from 3 independent experiments (n = 10-12/group, mean ± standard deviation). (C) Representative flow plots of the macrophage populations with the different treatments. All mice were infected with MHV68.ORF73β-lactamase. (D-F) Mice were infected as in A with 10^6^ PFU of MHV68.ORF73β-lactamase virus. Quantification of flow cytometric analysis of MHV68-infected PECs at day 2 post MHV68 infection. Data are pooled from 3 independent experiments (n = 10-12/group, mean ± standard deviation). (D) Quantification of the total number of MHV68-infected PECs. (E) Proportion of total MHV68-infected PECs for each treatment. (F) Number of MHV68-infected LPMs, SPMs, and B cells. (G) Proportion of MHV68-infected LPMs and SPMs out of the LPM and SPM populations, respectively. (H) Proportion of MHV68-infected LPMs and SPMs out of the LPM and SPM populations, respectively. 2-way ANOVA analysis compared proportion of LPMs to SPMs for each treatment. Each dot represents an individual mouse. (B, F, G, H) P-values, 2-way ANOVA, Tukey’s multiple comparisons * P ≤ 0.05, ** P ≤ 0.01, *** P ≤ 0.001, **** P ≤ 0.0001 (D, E) P-values, Ordinary 1-way ANOVA, Tukey’s multiple comparisons * P ≤ 0.05, ** P ≤ 0.01, *** P ≤ 0.001, **** P ≤ 0.0001.

We next asked whether expansion of permissive macrophages without concomitant parasite infection was sufficient to cause increased MHV68 infection. To test this question, we elicited an HP-independent LPM expansion with long-lasting IL-4 complexes (IL-4c) as previously described [13] or expansion of SPMs with thioglycolate broth [29–31]. We intraperitoneally injected IL-4c four days and two days before MHV68.ORF73β-lactamase infection, then treated once more with IL-4c before examining the PECs by flow cytometry (Fig 4A). Similarly, we chose to inject thioglycolate 2 days before MHV68 (Fig 4A) since most protocols call for macrophage collection 3 to 4 days post thioglycolate treatment [29–31]. This timeline causes SPM expansion before and after MHV68 infection, similar to what occurs with the LPMs and IL-4c treatment. As reported previously, IL-4c expanded the LPMs, but the numbers of SPMs and B cells were not affected (Fig 4B). Thioglycolate treatment caused expansion of the SPMs and initiated MDR of the LPMs (Fig 4B and 4C). The bimodal distribution of points in the thioglycolate expanded SPMs represents clustering of two different experiments and may represent variability between thioglycolate preparations.

We next examined the MHV68-infected cell populations. IL-4c treatment increased the total number of MHV68-infected PECs at day 2 post infection, but thioglycolate treatment did not (Fig 4D). IL-4c treated mice also had increased proportions of total infected PECs, compared to vehicle or thioglycolate treatments (Fig 4E). Further, the number of MHV68-infected LPMs was also increased with IL-4c treatment (Fig 4F), but there was no difference in the proportion of infected LPMs with IL-4c treatment (Fig 4G). IL-4c treatment did not significantly alter the numbers of infected SPMs or B cells (Fig 4F).

In contrast, there was not an increased number of infected total PECs with thioglycolate treatment even though SPMs were expanded (Fig 4D), nor was there an increase in the proportion of infected PECs (Fig 4E). Thioglycolate treatment did cause a significant increase in the number of infected SPMs, but it was much lower than the number of infected LPMs during IL-4c treatment (Fig 4F). There was also no change in the proportion of infected SPMs with thioglycolate treatment (Fig 4G). Thioglycolate treatment did not alter the number of infected B cells (Fig 4F).

These results were surprising since there were higher numbers of SPMs with thioglycolate treatment than there were LPMs with IL-4c treatment (Fig 4B). If expansion of an MHV68-permissive cell type is enough to cause increased infection, then the thioglycolate-expanded SPMs should have had higher numbers of infected cells. Since they did not, this suggests that LPMs are more permissive to MHV68 infection than SPMs. To quantify this, we examined the proportion of infected LPMs compared to SPMs for each treatment. We found that with all treatments, there was a higher proportion of infected LPMs than SPMs (Fig 4H). This mirrors the proportions of total infected PECs, which had increased proportions of infected PECs in the IL-4c group compared to the other two groups (Fig 4E). Overall, these findings demonstrate that HP-independent expansion of the LPM population is sufficient to increase MHV68 infection in PECs during acute MHV68 infection and that MHV68 preferentially infects LPMs. Moreover, these data suggest that the increased MHV68 infection we observed in LPMs during acute infection in mice with prior HP infection is caused by an increase in the reservoir of LPMs.

### Increased acute MHV68 infection in LPMs with HP coinfection is dependent on vitamin A and GATA6-mediated LPM expansion

**Fig 5:**
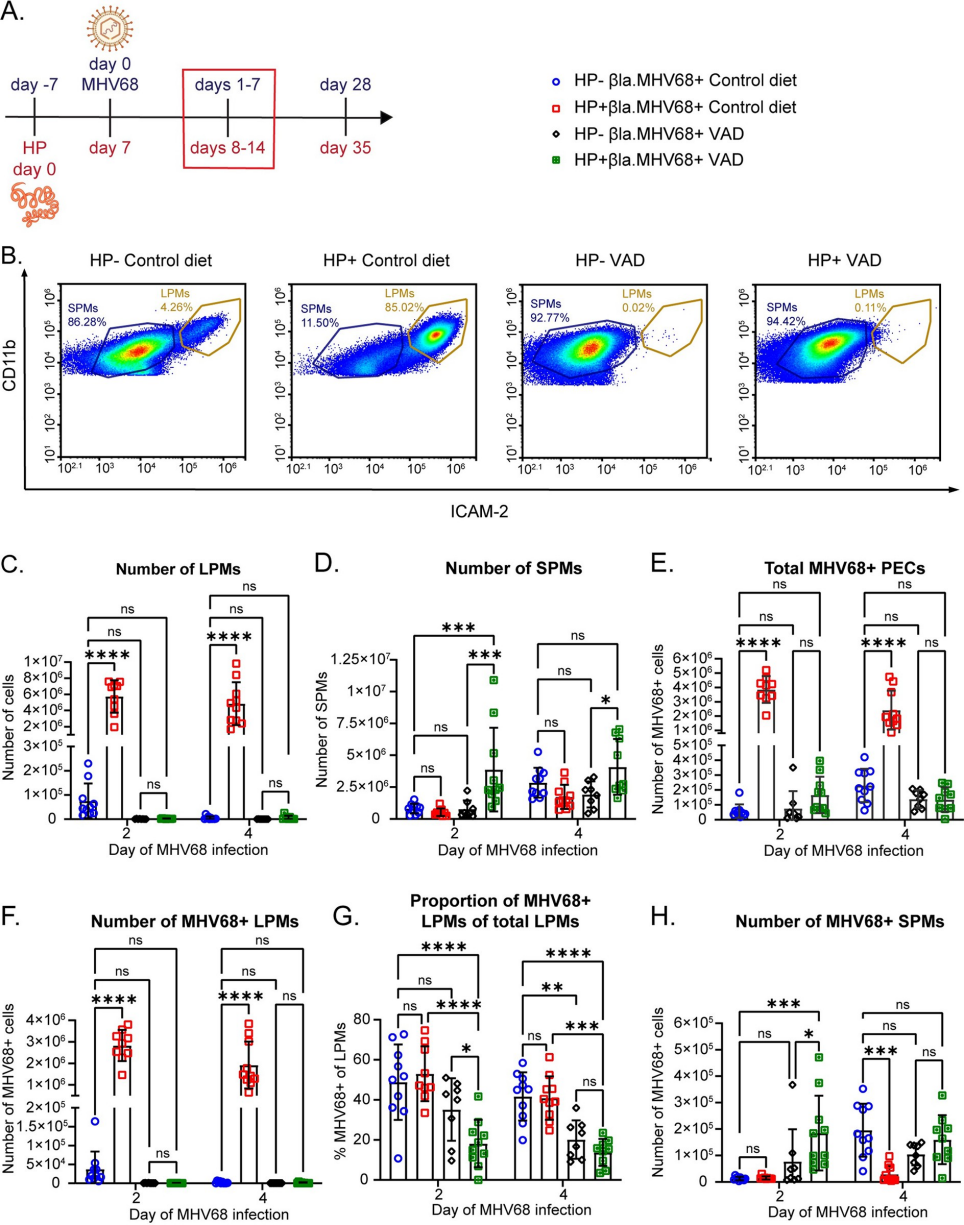
HP-induced increased MHV68 infection is dependent on vitamin A during the acute stage of MHV68 infection. Mice were raised on a vitamin A deficient diet or a control diet and infected with HP followed by the MHV68.ORF73β-lactamase reporter virus. PECs were collected on day 2 and day 4 of MHV68 infection. (A) Timeline of infections with HP by oral gavage and MHV68 by intraperitoneal injection (10^6^ plaque forming units (PFU)). The time points shown in (B-H) are outlined by a red box. Partially created in BioRender. (B) Representative flow plots of LPMs (ICAM-2^hi^ CD11b^hi^ CD19-) and SPMs (CD11b+ ICAM-2^lo^ CD19-) in each of the infection states and diets at day 2 of MHV68 infection. (C) Quantification of flow cytometric analysis of LPMs at days 2 and 4 of MHV68 infection. LPMs were gated as CD19- CD11b^hi^ ICAM-2^hi^. (D) Quantification of flow cytometric analysis of SPMs at days 2 and 4 of MHV68 infection. SPMs were gated as CD19- CD11b+ ICAM-2^lo^. Data are pooled from 2 independent experiments (n = 7-12/group, mean ± standard deviation). Each dot represents an individual mouse. P-values, 2-way ANOVA, Tukey’s multiple comparisons. (E-H) Quantification of flow cytometric analysis of MHV68-infected PECs at day 2 and 4 of MHV68 infection. Data are pooled from 2 independent experiments (n = 7-12/group, mean ± standard deviation). Each dot represents an individual mouse. (E) Total number of MHV68-infected PECs. (F) Number of MHV68-infected LPMs. (G) Proportion of MHV68-infected LPMs out of total LPMs. (H) Number of MHV68-infected SPMs. (C-F) P-values, 2-way ANOVA, Tukey’s multiple comparisons. * P ≤ 0.05, ** P ≤ 0.01, *** P ≤ 0.001, **** P ≤ 0.0001.

Since we saw that the majority of infected cells in HP/MHV68 coinfected mice were LPMs and we found that expansion of LPMs with IL-4c was sufficient to cause increased infection, we questioned if LPMs were necessary for the HP-induced increased infection. Vitamin A is required for LPMs; therefore, mice on a vitamin A deficient diet (VAD) are depleted of LPMs [32–34]. To generate mice that are vitamin A deficient, we put dams on the VAD or control diets 14 days after mating, and then maintained the offspring on their respective diets until euthanasia. VAD or control diet mice were infected first with HP, then infected with MHV68.ORF73β-lactamase one week later (Fig 5A). To confirm that the VAD diet does not affect HP infection, we counted HP at days 9 and 11 of HP infection from HP/MHV68 coinfected mice. There was no significant difference in worm burden at either timepoint between the control diet and VAD diet mice (S6 Fig). As expected, mice on the VAD diet lost the LPM population (Fig 5B and 5C). Further, during HP/MHV68 coinfection, we observed no expansion of LPMs in mice on the VAD diet, in contrast to HP/MHV68 coinfected mice on the control diet (Fig 5C). Interestingly, the number of SPMs was increased in HP/MHV68 coinfected mice on the VAD diet at days 2 and 4 of MHV68 infection (Fig 5D).

We next examined the MHV68-infected populations by using the MHV68.ORF73β-lactamase reporter virus. We found that HP/MHV68 coinfected mice on the VAD diet did not have increased numbers of total infected PECs, unlike mice on the control diet (Fig 5E). This same trend occurred with infected LPMs, as well: HP/MHV68 coinfected mice on the VAD diet did not have increased numbers of infected LPMs (Fig 5F). Further, the proportion of infected LPMs in HP/MHV68 coinfected mice on the VAD diet was decreased at day 2 of infection, even though the proportion of infected LPMs in MHV68-only infected mice was similar between the control diet and VAD diet groups (Fig 5G). At day 4 of infection, there was a significant decrease in the proportion of infected LPMs in MHV68-only VAD mice compared to MHV68-only control diet mice (Fig 5G). This suggests that vitamin A is important for MHV68 infection as the acute stage of infection progresses. We next examined the number of infected SPMs and found that there were increased numbers of infected SPMs in HP/MHV68 coinfected VAD mice at day 2 of MHV68 infection (Fig 5H). This corresponds to the increased number of SPMs in the VAD mice (Fig 5D). By day 4, there was no significant difference between the number of infected SPMs in the VAD groups (Fig 5H). Overall, these data demonstrate that LPMs and vitamin A are required for increased MHV68 infection at days 2 and 4 during parasite coinfection.

While the VAD data supports our hypothesis that LPMs are required for increased infection of PECs during coinfection with HP, vitamin A deficiency causes multiple defects in the immune system [32]. To confirm our findings, we used a macrophage-specific deletion of the transcription factor, GATA6 (Gata6^tm2.1Sad^; Csf1r^MeriCreMer^), to test whether LPMs are required for increased infection during coinfection. GATA6 is required for localization and normal transcriptional programming of LPMs [14,34]. We confirmed that Gata6^Δ Mac^ mice had reduced LPMs (F4/80-hi) compared to Gata6^flox/flox^ mice, and that other macrophage populations were not affected (S7A and S7B Fig). In addition, the F4/80hi LPMs did not significantly increase in HP/MHV68 coinfected Gata6^Δ Mac^ mice, demonstrating that GATA6 is required for LPM expansion during HP infection (S7B Fig).

Using the MHV68.ORF73β-lactamase system we measured the virally infected populations in the peritoneal cavity. As expected, we observed increased MHV68 infection in total PECs and LPMs at day 2 in HP/MHV68 coinfected Gata6^flox/flox^ mice, compared to MHV68-only infected Gata6^flox/flox^ mice, but we did not detect increased MHV68 infection in HP/MHV68 coinfected Gata6^Δ Mac^ mice (S7C and S3D Figs). This demonstrates that GATA6 is required for increased peritoneal infection during coinfection. There was no significant difference between the number of infected LPMs between the virus-only Gata6^flox/flox^ and Gata6^Δ Mac^ mice, suggesting that loss of GATA6 does not impact baseline MHV68 infection (S7D Fig). In addition, we found that there was no difference in proportion of infected F4/80hi LPMs between either of the Gata6^flox/flox^ groups, but that there was a decreased proportion of infected F4/80hi LPMs in the HP/MHV68 coinfected Gata6^Δ Mac^ group compared to the Gata6^Δ Mac^ virus-only group (S7E Fig). Overall, these results are in agreement with the VAD experiments and demonstrate that GATA6 is required for HP-induced LPM expansion and HP-induced increased infection.

### Early HP-independent expansion of LPMs and SPMs is not sufficient to cause increased latent infection

**Fig 6:**
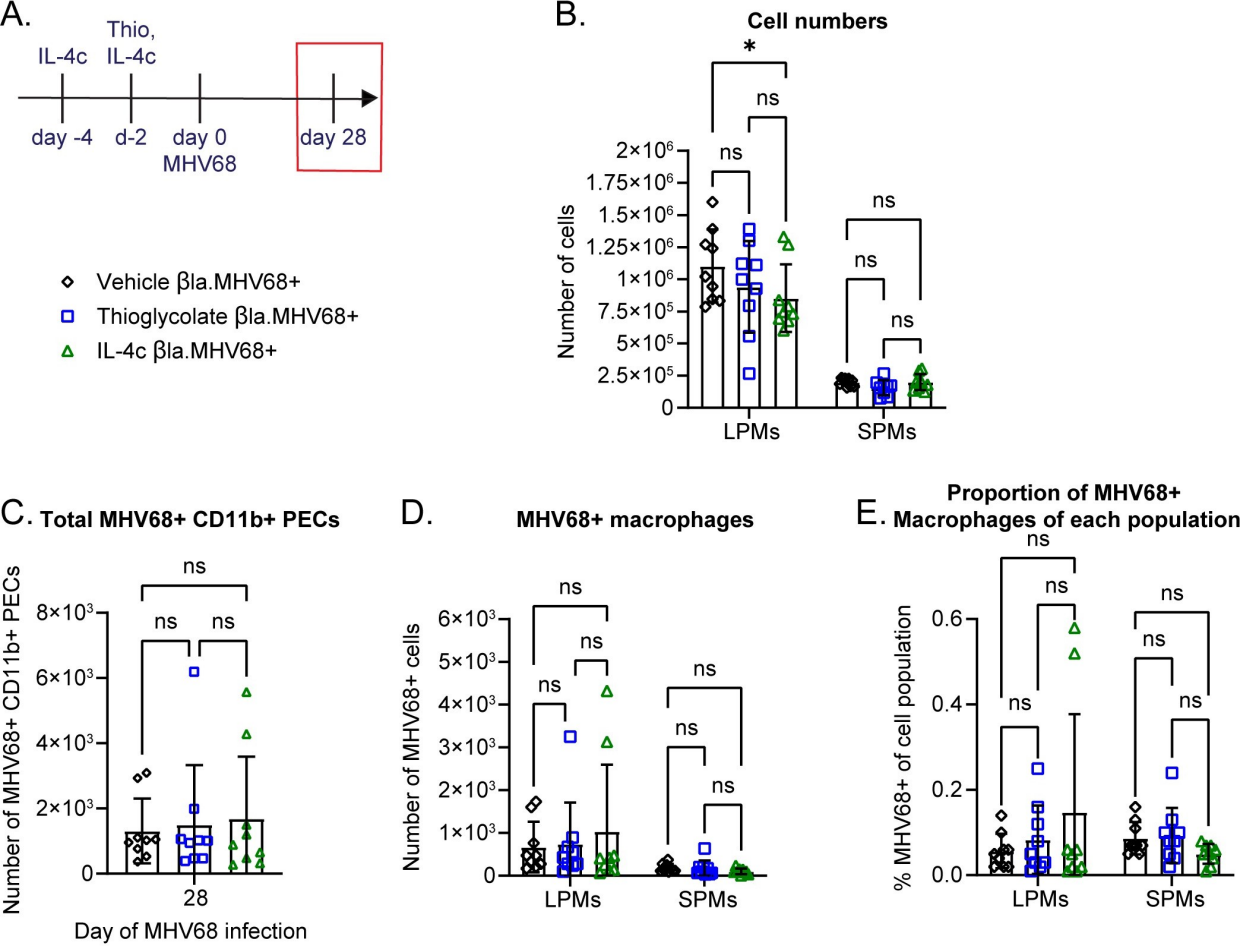
Early HP-independent expansion of LPMs and SPMs is not sufficient to cause increased latent infection. (A) Timeline of intraperitoneal IL-4 complex (IL-4c), thioglycolate (Thio) treatments and MHV68.ORF73β-lactamase intraperitoneal infection (10^6^ plaque forming units (PFU)). 5μg of IL-4 and 25 μg α-IL-4 were complexed and i.p. injected for LPM expansion. 3.8% sterile thioglycolate broth was i.p. injected to expand SPMs. The time point shown in (B-E) is outlined by a red box. (B) Quantification of flow cytometric analysis of LPMs and SPMs 28 days post MHV68 infection. LPMs were gated as CD19- CD11b^hi^ ICAM-2^hi^. SPMs were gated as CD19- CD11b+ ICAM-2^lo^. Data are pooled from 2 independent experiments (n = 9/group, mean ± standard deviation). (C-E) Mice were infected as in A with 10^6^ PFU of MHV68.ORF73β-lactamase virus. CD11b+ PECs were isolated to enrich for MHV68+ cells. Quantification of flow cytometric analysis of MHV68-infected PECs at day 28 post MHV68 infection. Data are pooled from 2 independent experiments (n = 9/group, mean ± standard deviation). (C) Quantification of the total number of MHV68-infected CD11b+ PECs. P-values, 1-way ANOVA, Tukey’s multiple comparisons. (D) Number of MHV68-infected LPMs and SPMs. (E) Proportion of MHV68-infected LPMs and SPMs of their respective parent populations. Each dot represents an individual mouse. (B, D, E) P-values, 2-way ANOVA, Tukey’s multiple comparisons * P ≤ 0.05, ** P ≤ 0.01, *** P ≤ 0.001, **** P ≤ 0.0001.

Now that we knew that LPM expansion was both sufficient and necessary for increased early MHV68 infection during parasite coinfection, we turned back to examine latency. We first asked whether LPM expansion at the time of MHV68 infection was sufficient to increase viral infection during latency. To address this, we utilized IL-4c expansion of the LPMs or thioglycolate expansion of SPMs as before. We treated mice with IL-4c twice or thioglycolate once to cause expansion of the LPMs and SPMs, respectively, before MHV68 was introduced (Fig 6A). We infected with MHV68.ORF73β-lactamase and waited for four weeks before examining latent infection using flow cytometry (Fig 6A).

We found that LPM numbers were lower in IL-4c treated mice than vehicle treated mice four weeks after infection (Fig 6B). There was no significant difference between the number of LPMs in thioglycolate treated mice and vehicle treated mice (Fig 6B). SPM numbers returned to homeostatic levels within four weeks post infection, in all groups (Fig 6B).

To enrich for MHV68 infected cells, we first isolated CD11b+ cells using positive selection beads before analyzing the PECs by flow cytometry. We found that the total number of infected CD11b+ PECs was not significantly different for any of the treatments (Fig 6C). Further, we found there was no difference in the number of infected LPMs or SPMs with any treatment (Fig 6D). The proportions of infected LPMs and SPMs were not significantly different with any treatments, either (Fig 6E). This suggests that the increased latent infection seen during HP/MHV68 coinfection requires the long-term maintenance of the LPM population, which is reduced during MHV68 infection alone (Fig 2B). Alternatively, HP infection may intrinsically alter the LPM population in a way that IL-4c treatment does not, which may promote increased MHV68 infection during latency. Overall, this data supports the hypothesis that maintenance of expanded LPM numbers during latency are required for increased latent MHV68 infection.

### HP-induced increased latent MHV68 infection requires vitamin A-mediated LPM expansion

**Fig 7:**
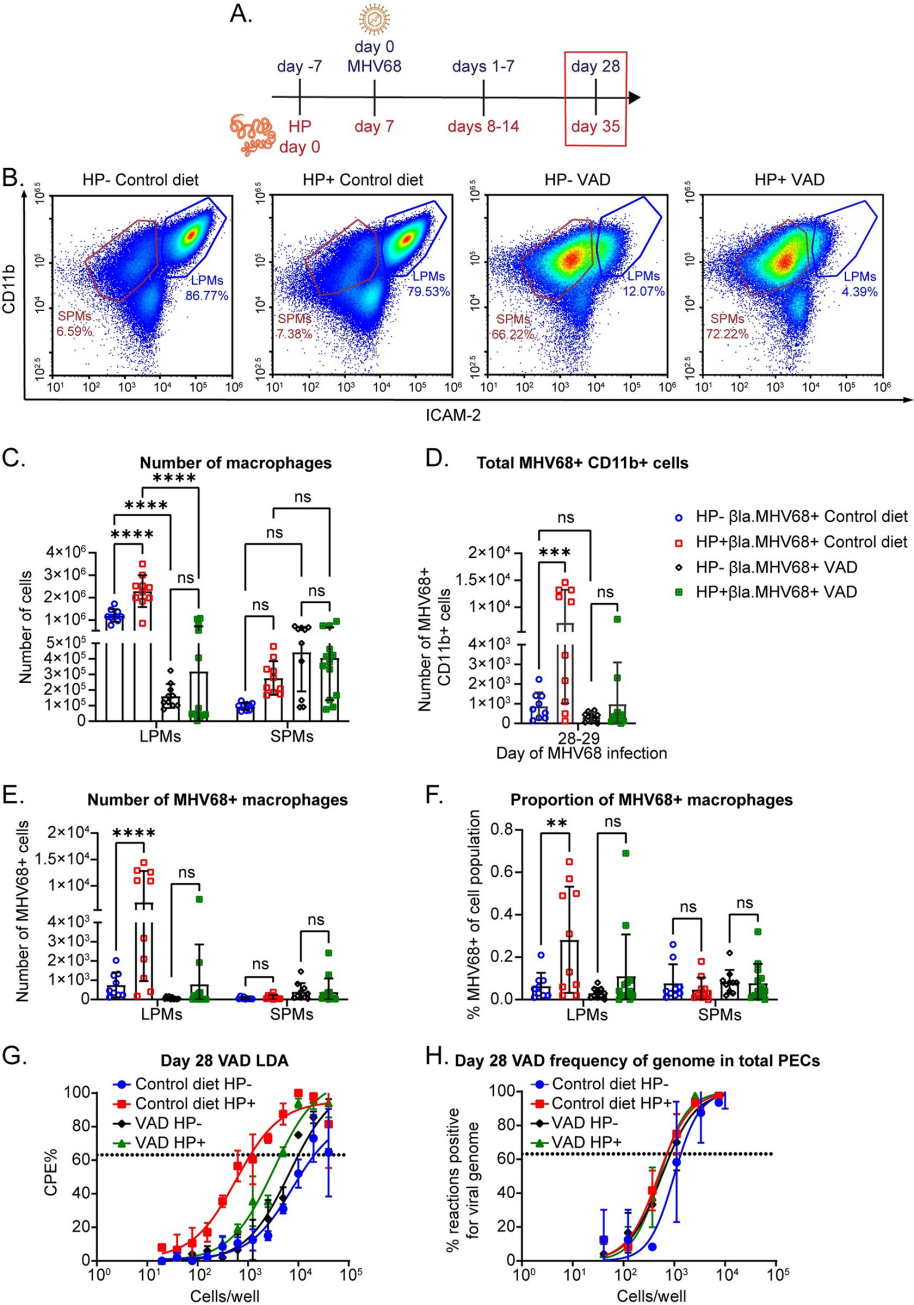
HP-induced increased latent MHV68 infection is dependent on vitamin A. (A) Mice were raised on a vitamin A deficient diet or a control diet and infected with HP followed by the MHV68.ORF73β-lactamase reporter virus, as in the timeline. PECs were collected on day 28 or 29 of MHV68 infection, as marked by the red box on the timeline. CD11b+ cells were isolated by MACs columns before staining. (B) Representative flow plots of the macrophage populations with the control diet or VAD at day 29 of MHV68 infection. Flow plots are from the single cell gate. (C) Quantification of flow cytometric analysis of LPMs and SPMs at day 29 of MHV68 infection. Data are pooled from 2 independent experiments (n = 9-13/group, mean ± standard deviation). Each dot represents an individual mouse. P-values, 2-way ANOVA, Tukey’s multiple comparisons. (D-F) Quantification of flow cytometric analysis of MHV68-infected CD11b+ PECs at day 28 or 29 of MHV68 infection. Data are pooled from 2 independent experiments (n = 9-13/group, mean ± standard deviation). Each dot represents an individual mouse. (D) Total number of MHV68-infected CD11b+ PECs. (E) Number of MHV68-infected macrophages. (F) Proportion of MHV68-infected cells out of the parent populations. (G-H) Mice on VAD or control diet were infected and PECs collected on day 28 of MHV68 infection. LDA’s and LD-PCR were performed on the total PECs to determine *ex vivo* reactivation (G) and frequency of viral genomes (H). Data are pooled from 2 independent experiments (3–5 mice pooled/group). Dotted line represents Poisson distribution. (C-F) P-values, 2-way ANOVA, Tukey’s multiple comparisons. * P ≤ 0.05, ** P ≤ 0.01, *** P ≤ 0.001, **** P ≤ 0.0001.

We next questioned whether the increased latent MHV68 infection induced by HP coinfection required LPMs and was dependent on vitamin A. We compared control diet and VAD diet mice 28–29 days post MHV68 infection (Fig 7A). We first verified that the VAD diet depleted LPMs by examining ICAM-2^hi^ CD11b^hi^ LPMs by flow cytometry (Fig 7B). We observed decreased numbers of LPMs in both MHV68-only mice and HP/MHV68 coinfected mice on the VAD diet (Fig 7B and 7C). SPMs were equivalent for all groups (Fig 7C). To test whether VAD affects chronic HP infection, we measured fecundity and worm burden at day 35 of HP infection in HP/MHV68 coinfected mice. Worm burden and fecundity were equivalent between the two diets (S8A Fig), so chronic HP infection is not affected by loss of vitamin A.

We next used the MHV68.ORF73β-lactamase virus to quantify the number of infected cells. As before, we selected CD11b+ PECs to enhance our detection of latently infected cells. We found that HP/MHV68 coinfected mice on the control diet had a higher number of infected CD11b+ PECs than MHV68-only mice on the control diet (Fig 7D). In contrast, we observed no increase in latent infection with HP coinfection in mice on the VAD diet (Fig 7D). Importantly, because there was no difference in MHV68 infection in MHV68-only mice on the control diet and VAD diet (Fig 7D), we can conclude that loss of vitamin A did not disrupt baseline MHV68 latency establishment.

Next, we quantified the number of infected macrophages. There was an increased number and proportion of infected LPMs in HP/MHV68 coinfected mice on the control diet, compared to virus-only mice on the control diet (Fig 7E and 7F). In contrast, there was no significant difference in the number or proportion of infected LPMs between HP/MHV68 coinfected and virus-only VAD diet mice (Fig 7E and 7F). The number and proportion of infected SPMs between virus-only and HP/MHV68 coinfected mice had no significant differences for either diet (Fig 7E and 7F). Overall, this data demonstrates that the increased latency in LPMs during coinfection is dependent on vitamin A. It is still unclear whether this dependency on vitamin A is because the HP-induced increase in the number of LPMs is required, or because vitamin A signaling plays a role in latency establishment or maintenance.

### Coinfection with *H*. *polygyrus* increases *ex vivo* MHV68 reactivation in a vitamin A dependent manner

Since we demonstrated that the helminth-induced increase in infection was dependent on vitamin A, we questioned whether that was true for the helminth-induced reactivation. We followed the infection protocol in Fig 7A and analyzed viral reactivation by explanting peritoneal exudate cells for a limiting dilution viral reactivation assay. HP/MHV68 coinfected mice on the control diet had an 18-fold increase in reactivation compared to the virus-only control diet mice (1 out of 20,930 in MHV68-only versus 1 out of 1124 in coinfected) (Fig 7G). In contrast, HP/MHV68 coinfected mice on the VAD diet had only a 2-fold helminth-induced increase in reactivation compared to virus-only mice on the VAD diet (1 out of 10,089 in MHV68-only VAD versus 1 out of 4,479 in coinfected VAD) (Fig 7G). Disruption of samples confirmed that the virus was latent and there was no *in vivo* reactivation in any of the groups (S8B Fig), which demonstrates that the immune response in VAD mice was sufficient to maintain latency of MHV68.

To ensure that loss of vitamin A did not disrupt the establishment of latency, we performed LD-PCR on total PECs to detect viral genome and confirmed that latency establishment was similar in the PECs for all groups (Fig 7H). HP/MHV68 coinfected mice on the control diet had a 2-fold increase in frequency of genomes in total PECs compared to MHV68-only mice on the control diet (1 out of 1,347 in MHV68-only versus 1 out of 714 in coinfected) (Fig 7H). Both VAD groups had equivalent frequencies of genome (1 out of 844 in MHV68-only VAD versus 1 out of 725 in coinfected VAD) (Fig 7H). Our results from the LDA and LD-PCR suggest that HP-induced reactivation is dependent on vitamin A, but VAD does disrupt the proportions of infected LPMs and SPMs during HP/MHV68 coinfection, so that there are similar numbers of LPMs and SPMs (Fig 7C). It is possible that SPMs reactivate MHV68 less efficiently than LPMs, and that the similar proportions of SPMs and LPMs in mice on the VAD diet is the cause of reduced reactivation in coinfected VAD mice. While we have not directly tested this hypothesis, we still can conclude that vitamin A plays a role in reactivation of MHV68 during coinfection when we compare the frequency of reactivation to the frequency of latency. Even with the increased latency of the coinfected control diet mice considered (18-fold increase in reactivation compared to 2-fold increase in latency), the increase in reactivation for the HP/MHV68 coinfected mice on the control diet was much higher than the increase in reactivation of the coinfected VAD mice (2-fold increase in reactivation compared to no change in latency). Since there is a higher fold-change in reactivation compared to latency for the control diet mice, but not a similar increased fold change for the VAD groups, we conclude that vitamin A is required for the increased reactivation induced by coinfection. Overall, these results demonstrate that coinfection with a helminth induces increased *ex vivo* reactivation of MHV68 in a vitamin A dependent manner. Based on these results, we cannot determine whether the loss of LPMs from the VAD diet or if the loss of vitamin A alone is affecting the HP-induced increased reactivation.

## Discussion

**Fig 8:**
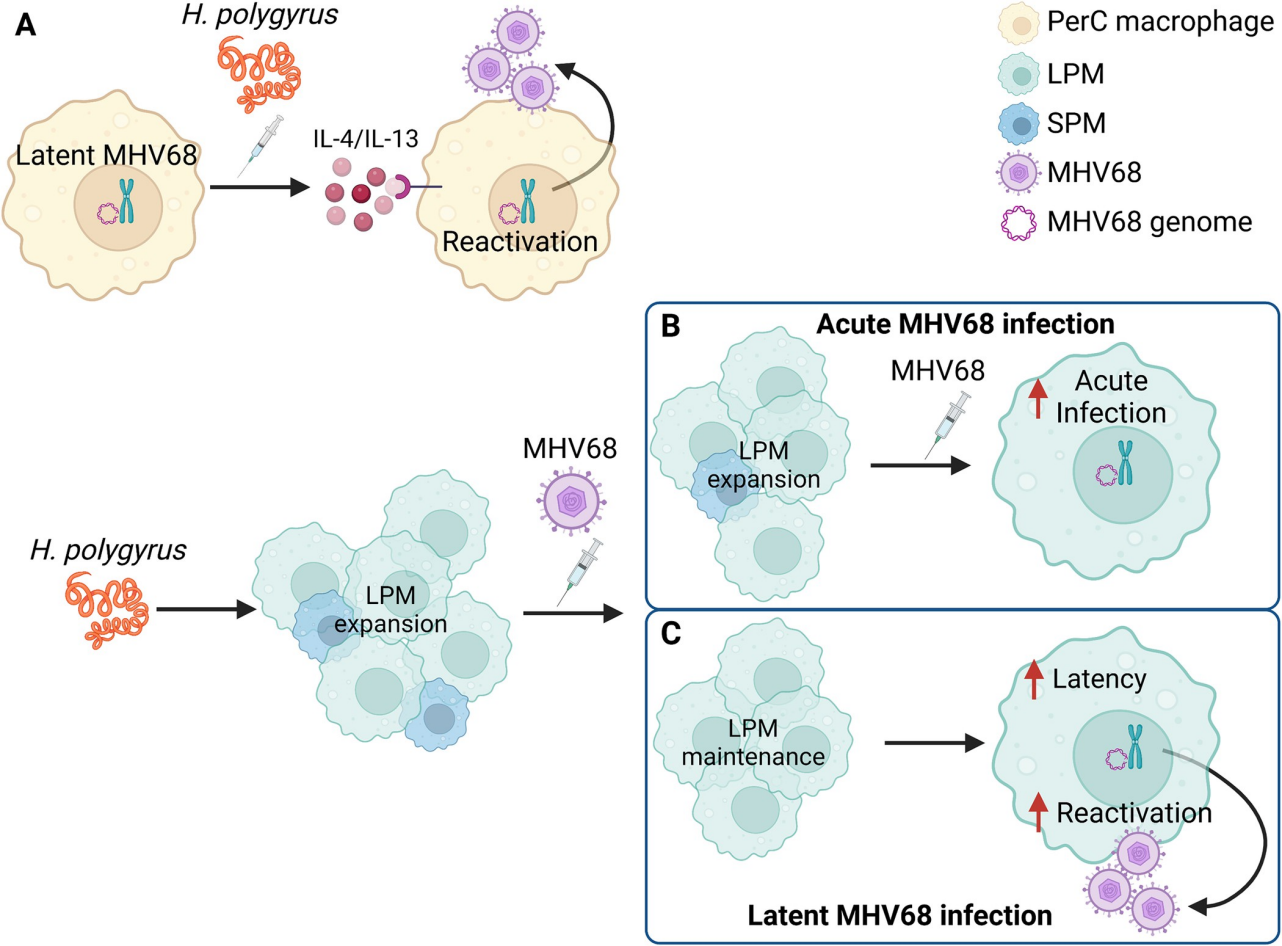
Comparison of MHV68/HP and HP/MHV68 coinfection models and mechanisms. (A) Model of HP challenge during MHV68 latency inducing reactivation through IL-4 and IL-13 signaling. (B) HP infection prior to MHV68 coinfection. HP-induced increased acute MHV68 infection is driven by LPM expansion. (C) HP infection prior to MHV68 infection. HP infection maintains the LPM population and possibly induces LPM-intrinsic changes that drive increased latent infection and increased *ex vivo* reactivation. Model created in BioRender.

The purpose of this study was to determine the impact of intestinal parasite infection on MHV68 infection and latency. While our previous study focused on reactivation of MHV68 when HP infection occurs during latency (Fig 8A) [7], in this study we demonstrated that when HP is infected first it exacerbates all stages of MHV68 infection. Our results reported here compared with our previous study on HP/MHV68 coinfection reveal that the timing of coinfection dictates mechanism. We previously discovered that when mice with a latent MHV68 infection were challenged with HP four to six weeks after the initial viral infection, the HP infection induced reactivation *in vivo*. This reactivation required STAT6 signaling, suggesting that IL-4/IL-13 produced in response to the HP infection promoted virus reactivation (Fig 8A). In contrast, in this study we first infected mice with HP and challenged with MHV68 seven days later. While there is increased *ex vivo* reactivation of the virus in HP-infected mice, the mechanism did not require STAT6 signaling. Together, these two systems reveal that the order and timing of coinfection are critical variables in the determination of mechanism.

During the acute stage, MHV68 infection of the PECs was increased in HP/MHV68 coinfected mice. The increased acute infection primarily occurred in LPMs in vitamin A- and GATA6-dependent manners. However, the increased infection did not result in increased replication, suggesting that the virus was latent. We demonstrated that the HP-independent expansion of LPMs is sufficient to increase acute MHV68 infection in LPMs, suggesting that increased acute infection during HP/MHV68 coinfection is driven by increased LPM numbers (Fig 8B). However, expansion of the SPM population of macrophages was not sufficient to increase MHV68 acute infection, perhaps indicating that LPMs are a preferred site of MHV68 infection. TIM-4+ LPMs are known to clear apoptotic cells in the peritoneal cavity, and they downregulate the CpG DNA sensor, toll like receptor (TLR)-9, perhaps to minimize inflammation [35]. TLR9 plays a critical role in control of MHV68 [36]. It was reported that MHV68 has fewer TRL9 immunostimulatory motifs [37], suggesting that MHV68 evolved to evade TRL9 recognition. Altogether, this suggests that LPMs are more permissive to MHV68 infection. It is also possible that the less inflammatory environment in LPMs promotes latency establishment rather than replication of MHV68.

During latency, there were increased numbers and proportions of infected LPMs in HP/MHV68 coinfected mice, which were also dependent on vitamin A. In contrast to acute infection, we found the IL-4 -mediated expansion of LPMs during acute infection was not sufficient to cause increased latent infection, perhaps because LPM numbers returned to homeostatic levels by day 28 after IL-4 treatment. This indicates that HP maintenance of the LPM population or intrinsic changes to the LPM population during HP/MHV68 coinfection is required for increased reactivation (Fig 8C). In future experiments it would be interesting to further manipulate the timing of coinfection by infecting with MHV68 at later intervals after HP infection.

Coinfected mice had vitamin A dependent increased *ex vivo* reactivation, a measure of how easily the virus can reactivate, which likely requires LPMs (Fig 8C). This raises two possibilities: HP-induced reactivation could be dependent on vitamin A signaling or HP-induced reactivation could be dependent on the vitamin A-dependent LPM population. Vitamin A is a precursor to the biologically active retinoic acid, which was shown to reactivate MHV68 in macrophages by removal of corepressors from the ORF50 promoters [5]. Alternatively, the number of infected LPMs, which require vitamin A for GATA6 expression and expansion [34], could be driving the HP-induced reactivation. We think this possibility is less likely since the fold increase of the frequency of MHV68 genomes in LPMs (Fig 2H) is less than the fold increase of the frequency of reactivation (Fig 1B). We observed a 7-fold increase in the frequency of genome in LPMs from HP/MHV68 coinfected mice compared to MHV68-only mice, but a 16-fold increase in *ex vivo* reactivation in HP/MHV68 coinfected mice. This suggests that increased reactivation is not simply caused by a higher proportion of infected LPMs in the HP/MHV68 coinfected mice but is truly a reactivation phenotype.

An intriguing observation is that helminth infection appears to buffer the macrophage disappearance reaction (MDR). Our data agree with other reports that parasites expand LPMs [13,14,33]. In contrast, bacterial and viral infections trigger MDR in the peritoneal cavity [24,28]. During MDR, LPMs migrate to the omentum [34], the periphery of the peritoneal cavity, or form clots [28]. Viral infection in the peritoneal cavity also causes MDR, because analysis of the peritoneal cell populations during acute replication of MHV68 showed that LPMs were depleted by day 8 of infection and reappeared by day 14 [24]. This is consistent with our observations, where MHV68-only infected mice lose LPMs during infection but have a prominent LPM population during latency. Perhaps more intriguing, is the fact that coinfected mice did not lose their LPM population, which indicates that the helminth infection helps maintain the LPM population during acute inflammation in the peritoneal cavity. Humans, unless they currently live in developed countries, are infected with parasites throughout their lives. Further, while certain peritoneal cavity macrophage functions have been conserved throughout evolution, it is unknown if parasite-induced proliferation of human peritoneal cavity macrophages occurs [38]. More studies of human LPM biology in the context of infections are needed. In individuals with chronic intestinal parasite infection, tissue resident macrophage populations may be maintained, which may confer susceptibility or resistance to infections of other tissues. There is evidence that LPMs help control bacterial peritoneal infections [28,39], so helminth induced maintenance of this population likely improves survival during bacterial peritonitis.

Overall, we demonstrated that pre-infecting mice with an intestinal helminth increased MHV68 infection in LPMs during acute replication and increased LPM infection during latency. HP/MHV68 infection also increased *ex vivo* reactivation of the virus, which is lost with VAD-depletion of LPMs. Notably, the increased MHV68 reactivation with preexisting HP infection did not require STAT6 signaling, in contrast to our previous work where HP challenge occurred after MHV68 infection. Overall, our study demonstrates the timing of coinfection is an important variable in determining the mechanism for parasite mediated reactivation of chronic herpesvirus infection.

## Methods

### Ethics statement

All procedures were approved by the University of Texas Southwestern Medical Center Institutional Animal Care and Use Committee (IACUC) under protocol number 2015–100990.

### Mice

Animals were bred and housed in pathogen-specific free facilities. Mice were bred in a barrier facility and experiments were performed in a conventional facility. Unless noted, C57BL/6J mice were used in the experiments. *Stat6*^-/-^ (B6.129S2(C)-*Stat6*^*tm1Gru*^/J, stock number 005977) [40] mice were purchased from Jackson Labs and bred to C57BL/6 mice to generate littermate controls or maintained in a *Stat6*^-/-^ homozygous cross. *Gata6*^f/f^ (*Gata6*^*tm2*.*1Sad*^/J, Jackson stock number 008196) [41] and CD115 Cre-ER (Tg(*Csf1r*-cre/Esr1*)1Jwp/J, Jackson stock number 019098) [28,42,43] mice were a gracious gift from Dr. Gwendalyn Randolph. *Gata6*^f/f^ mice were crossed with CD115 Cre-ER mice, so that each litter produced cre- and cre+ littermate controls. To delete *Gata6* from macrophages and monocytes, tamoxifen (Sigma # T5648) was dissolved at 20 mg/mL in autoclaved corn oil (Sigma # C8267) by shaking overnight at 37 degrees C in the dark. Mice were orally gavaged with 4 mg of tamoxifen (200 μL of 20 mg/mL stock) dissolved in corn oil for three consecutive days. 5 μg of IL-4 (Peprotech, AF-214-14) and 25 μg of α-IL-4 (BioXcell, clone 11B11) were complexed prior to intraperitoneal injection into mice. For thioglycolate treatment, mice were injected with 1 mL of 3.8% sterile thioglycolate broth (Sigma, T9032). Experiments utilized a mix of both male and female mice. Mice were aged 5–14 weeks old at the start of infections.

### Animal infections

Mice were orally gavaged with 100 L3 *H*. *polygyrus* one week before infection with MHV68. Mice were infected with 1 x 10^6^ pfu of MHV68 (WUSM strain) intraperitoneally. MHV68.ORF73β-lactamase was a gift from Dr. Scott Tibbetts and has been previously described [25].

### *H*. *polygyrus* (HP) maintenance

C57BL/6 or *Stat6*^-/-^ mice were infected with 200–400 L3 HP by oral gavage. Two weeks after infection, grates were placed in the bottom of the cages and feces were collected for 3–5 days. Feces were plated with washed and autoclaved deactivated charcoal (C2764, Sigma). 7–10 days after fecal plating, L3 larva were collected using a Baermann apparatus as previously described [9]. Larva were stored at 4°C in PBS for up to 6 months.

### HP worm burden and fecundity

Small intestines were collected at the time of euthanasia. Small intestines were cut open longitudinally and incubated at 37°C for 1–5 hours in PBS. Adult worms were counted by eye. For fecundity, fecal pellets were collected from the colon at the time of euthanasia. The fecal pellets were suspended (or dissolved) in water. An equal volume of saturated sodium chloride (Sigma) was added and eggs were enumerated on a McMaster slide (Electron Microscopy Sciences).

### Vitamin A deficient diet model

Female C57BL/6 breeders were given regular chow until day 14 of gestation, as described [32]. The females were then given either control diet (Teklad, TD.09838) or vitamin A deficient diet (Teklad, TD.09839) until the pups were weaned. Weaned pups were fed their respective diets throughout the experiment until they were sacrificed. Mice were housed with Sani-chip bedding to avoid any uncontrolled vitamin A intake.

### MHV68 Plaque assays

PECs were harvested by peritoneal lavage with 10 ml of complete D5 ((DMEM (Corning), 5% FBS (Biowest), 2mM L-glutamine (Gibco), 1% HEPES (Corning), 10 U/mL Penicillin and 10 μg/mL streptomycin sulfate (Corning)) and washed once. The PECs were resuspended in 1 ml of D5, then were homogenized with 1 mm zirconia beads (BioSpec Products) in a Precellys 24 (Bertin Instruments) at 5000 rpm for 1 min. 10-fold serial dilutions were plated on 3T12s. Virus was absorbed for 1 hour at 37 degrees C before 1% (w/v) methylcellulose (Sigma-Aldrich) (10 g methylcellulose in 1L MEM (Corning)) was added as an overlay. Plates were incubated at 37 degrees C in 5% CO_2_ for 7 days before fixing with 2% formaldehyde and staining with 0.1% crystal violet (Sigma-Aldrich).

### Limiting dilution assays (LDAs)

Limiting dilution assays were performed as previously described [18]. Briefly, peritoneal exudate cells (PECs) were collected by lavage in 10 ml of complete D10 (DMEM (Corning), 10% FBS (Biowest), 2mM L-glutamine (Gibco), 1% HEPES (Corning), 10 U/mL Penicillin and 10 μg/mL streptomycin sulfate (Corning)) and washed once. Cells were counted. In addition, spleens were collected and homogenized in glass tissue disrupters. Splenocytes were RBC lysed (ACK lysing buffer, Gibco), washed, and counted. 2 x 10^6^ PECs or 5 x 10^6^ splenocytes were used for the 0 dilution. Two-fold dilutions were performed and plated on C57BL/6 mouse embryonic fibroblasts. For disrupted samples, 2 x 10^6^ PECs or 5 x 10^6^ splenocytes were homogenized in a Precellys 24 (Bertin Instruments) at 6000 rpm for 1 min. Two-fold dilutions were plated of the disrupted samples. The assay was scored for cytopathic effect three weeks after plating.

### Limiting dilution (LD)-PCR

Samples from the LDAs were frozen and utilized for LD-PCR. LD-PCR was performed as previously described [20]. Briefly, cells were counted and 8 x 10^5^ cells (including dead cells) were used to make 3-fold dilutions. The dilutions were used in nested-PCR reactions for ORF72 of MHV68. The proportion of positive reactions was used to quantify the frequency of latency. For samples that were sorted before LD-PCR, 3 x 10^5^ cells starting cells were used. Primers for ORF72: Orf72in F (TGTCAGCTGTTGTTGCTCCT); Orf72in R (CTCCGTCAGGATAACAACGTCT); Orf72out F (GAGATCTGTACTCAGGCACCTGT); Orf72out R (GGATTTCTTGACAGCTCCCTGT).

### Flow cytometry

Peritoneal exudate cells (PECs) were collected by lavage with 10 mL of flow buffer (1% FBS in PBS (Corning)). PECs were washed once, then Fc receptors were blocked with α-CD16/32 (Biolegend). The following antibodies were used in various experiments: Alexafluor 647-α-CD102 (3C4 (MIC2/4), Biolegend), APC-Cy7-α-CD19 (1D3, BD Biosciences), redFluor 710-α-CD11b (M1/70, Tonbo), Fitc-α-CD11b (M1/70, BD Biosciences), PE-Cy7-α-CD19 (1D3, Tonbo), Violetfluor450-α-CD3 (17A2, Tonbo), and BV711-α-Siglec F (E50-2440, BD Biosciences). Cells were fixed with 2% formaldehyde (VWR). Samples were run on a Novoctye 3000 (ACEA Biosciences) and analyzed with either Flowjo (Tree Star, Inc, San Carols, CA) or NovoExpress (version 1.5.0, Agilent Technologies, Inc.).

### MHV68.β-lactamase detection by flow cytometry

PECs were collected and washed once. For latency experiments, cells were counted and CD11b+ cells were selected by MACS column purification using the CD11b MicroBead kit and LS columns (Miltenyi Biotec). Total PECs were used for MHV68 acute infection experiments. CD11b+ cells or whole PECs were Fc blocked with α-CD16/32 (Biolegend) and stained with Alexafluor 647-α-CD102 (3C4 (MIC2/4), Biolegend), APC-Cy7-α-CD19 (1D3, BD Biosciences), redFluor 710-α-CD11b (M1/70, Tonbo) or APC-Cy7-α-CD11b (M1/70, Biolegend), and R718-α-CD102 (3C4 (MIC2/4), BD Biosciences). Cells were counted again and resuspended in 3 x 10^6^ cells/mL before adding CC4F-AM (LiveBLAzer FRET-B/G Loading Kit with CCF4-AM, ThermoFisher Scientific). Cells were incubated for 1 hour, then washed with PBS and fixed with 2% formaldehyde. Samples were immediately run on a Novocyte 3000 (ACEA Biosciences).

### Flow sorting

Three to four mice were pooled for each sample. PECs were collected and washed. Cells were counted and CD11b+ cells were selected by MACS column purification using the CD11b MicroBead kit and LS columns (Miltenyi Biotec). CD11b+ cells were stained with Fc block α-CD16/32 (Biolegend) and then PE-Cy7-α-CD19 (1D3, Tonbo), Fitc-α-CD11b (M1/70, BD Biosciences), BV510-α-F4/80 (BM8, Biolegend), BV711-α-Siglec F (E50-2440, BD Biosciences), Violetfluor 450-α-Ly-6G (GR1) (RB6-8B5, Tonbo), and Alexafluor 647-α-CD102 (3C4 (MIC2/4), Biolegend). Samples were run on a BD FACS Aria II SORP and collected in D10 media (for LD-PCR). Afterward, the samples were counted and frozen for LD-PCR (in media).

### Statistics

Analysis was performed with GraphPad Prism 9.3.1. An unpaired t-test was used to analyze data with two groups. One-way or 2-way ANOVA was used to analyze data with more than two groups, with Tukey’s multiple comparisons. Linear regression analysis was used to create the curves for the LDA and LD-PCR graphs. Frequencies of reactivation or latent genome were calculated using the linear regression equations provided by GraphPad Prism. Error bars depict the standard deviation. Significance was considered significant when *P* ≤ 0.05.

## Supporting information

S1 Fig Preformed LDAs and impact of MHV68 on chronic HP infection. (A-D) HP-infected or uninfected mice were challenged with 10^6^ PFU of MHV68 i.p. PECs and/or splenocytes were isolated at day 28–31 of MHV68 infection. (A) C57BL/6 PECs were collected at day 28–31 of MHV68 infection for LDAs. PECs from (Fig 1B) were disrupted before plating to detect preformed virus. Data pooled from 4 independent experiments (3 mice pooled/group). Dotted line represents Poisson distribution. (B) C57BL/6 splenocytes were collected at day 28–31 of MHV68 infection for LDAs. Splenocytes from (Fig 1C) were disrupted before plating to detect preformed virus. Data pooled from 4 independent experiments (3 mice pooled/group). Dotted line represents Poisson distribution. (C) Adult worm burden and fecundity of HP in helminth-only and coinfected mice at days 35 and 36 of HP infection, which correspond to days 28 and 29 of MHV68 infection. Data are pooled from 2 independent experiments (n = 8 mice/group, mean± standard deviation). Each dot represents an individual mouse. P-values, Unpaired t-test. * P ≤ 0.05, ** P ≤ 0.01, *** P ≤ 0.001, **** P ≤ 0.0001. (D) Littermate controls of *Stat6*^*-/-*^ PECs from (Fig 1D) were disrupted before plating to detect preformed virus. Data are pooled from 2 independent experiments (3 mice pooled/group). Dotted line represents Poisson distribution.
(TIF)

S2 Fig Gating strategy for MHV68+ PECs during latency. CD11b+ cells were isolated by microbeads before flow to enrich for MHV68+ cells.
(TIF)

S3 Fig Gating controls for βla.MHV68+ cells. (A) Gates for βla.MHV68+ cells in virus-only mice were set on populations from WT MHV68+ mice. WT MHV68 does not produce signal with the CCF4-AM substrate. (B) Gates for βla.MHV68+ cells in HP/MHV68 coinfected mice were set on populations from HP/WT MHV68+ coinfected mice. The same strategy was used for every βla.MHV68 experiment.
(TIF)

S4 Fig Gating strategy for LPMs. (TIF)

S5 Fig Gating strategy for MHV68+ PECs during acute replication. (TIF)

S6 Fig Effect of VAD on early HP infection. (A) Mice were raised on a vitamin A deficient diet or a control diet and infected with HP and the MHV68.ORF73β-lactamase reporter virus, as in Fig 5. Worm burden of HP at days 9 and 11 of HP infection, which correspond to days 2 and 4 of MHV68 infection. Data are pooled from 2 independent experiments (7–12 mice/group, mean± standard deviation). Each dot represents an individual mouse. P-values, 2-way ANOVA, Tukey’s multiple comparisons. * P ≤ 0.05, ** P ≤ 0.01, *** P ≤ 0.001, **** P ≤ 0.0001.
(TIF)

S7 Fig Increased MHV68 infection during HP coinfection requires GATA6-mediated LPM expansion during acute MHV68 infection. (A-E) Tamoxifen treated Gata6^flox/flox^ and Gata6^Δ Mac^ mice were infected with the MHV68.ORF73β-lactamase reporter virus, as in Fig 3. PECs were collected at day 2 of MHV68 infection for flow analysis. (A) Representative flow plots of macrophage gating at day 2 of MHV68 infection. (B-E) Quantification of flow cytometric analysis of MHV68-infected PECs at day 2 of MHV68 infection. Data are pooled from 2 independent experiments (n = 9-10/group, mean ± standard deviation). Each dot represents an individual mouse. (B) Number of macrophages. (C) Total number of MHV68-infected PECs. (D) Number of MHV68-infected macrophages. (E) Proportion of MHV68-infected macrophages out of the respective parent macrophage populations. P-values, 2-way ANOVA, Tukey’s multiple comparisons. * P ≤ 0.05, ** P ≤ 0.01, *** P ≤ 0.001, **** P ≤ 0.0001.
(TIF)

S8 Fig Effect of VAD on chronic HP infection. (A-B) Mice were raised on a vitamin A deficient diet or a control diet and infected with HP and the MHV68.ORF73β-lactamase reporter virus, as in Fig 7. (A) Worm burden and fecundity of HP at days 35 and 36 of HP infection, which correspond to days 28 and 29 of MHV68 infection. Data are pooled from 2 independent experiments (4–6 mice/group, mean± standard deviation). Each dot represents an individual mouse. P-values, unpaired t-test. * P ≤ 0.05, ** P ≤ 0.01, *** P ≤ 0.001, **** P ≤ 0.0001. (B) PECs from (Fig 7G) were disrupted before plating to detect preformed virus. Data are pooled from 2 independent experiments (3–5 mice pooled/group). Dotted line represents Poisson distribution.
(TIF)
