# Supplementary figures and images for: Preexisting helminth challenge exacerbates infection and reactivation of gammaherpesvirus in tissue resident macrophages
Source: PLoS Pathog. 2023 Oct 17;19(10):e1011691. doi: 10.1371/journal.ppat.1011691 (PMC10581490; doi:10.1371/journal.ppat.1011691)

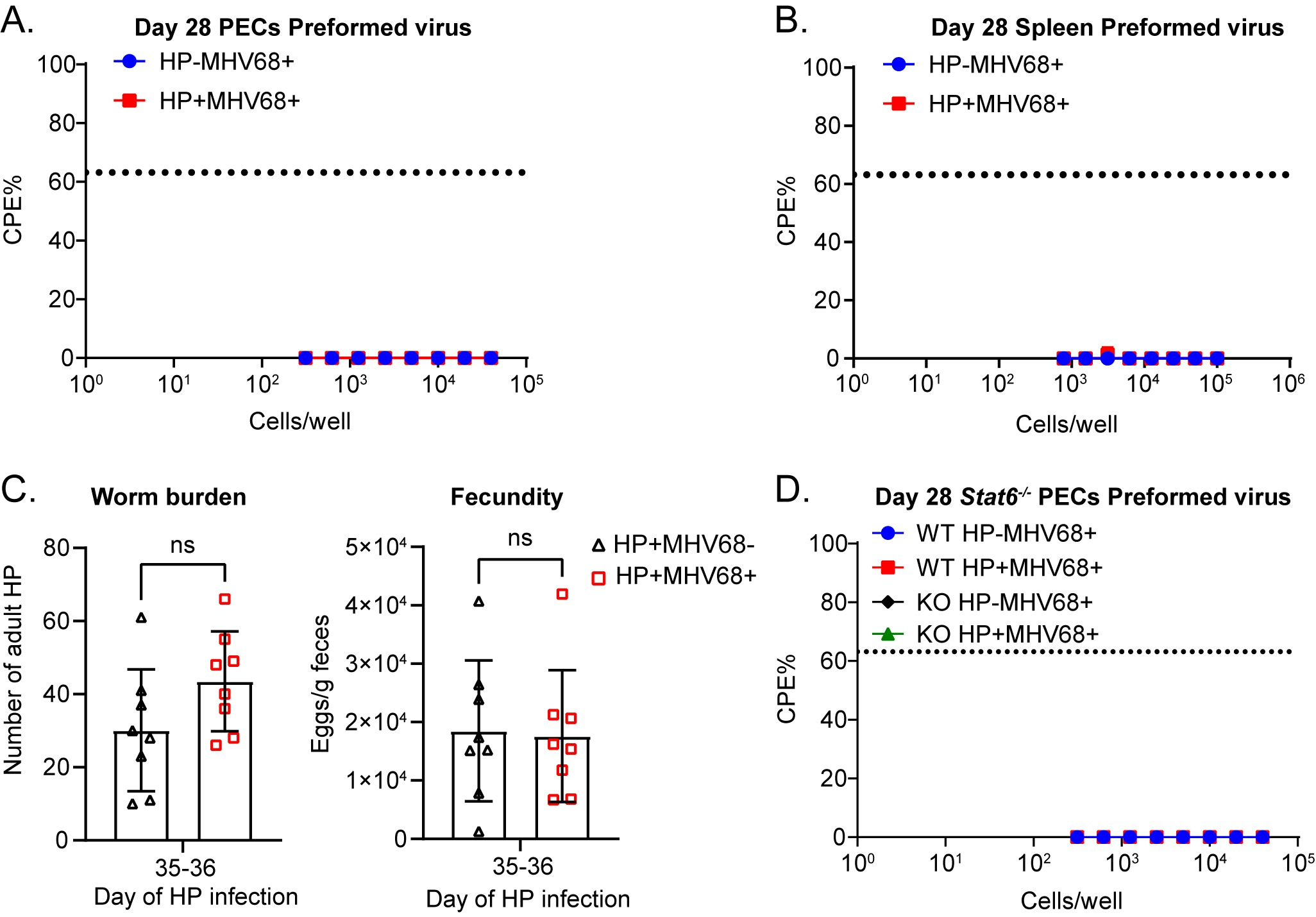

Supplement: S1 Fig — (A-D) HP-infected or uninfected mice were challenged with 106 PFU of MHV68 i.p. PECs and/or splenocytes were isolated at day 28–31 of MHV68 infection. (A) C57BL/6 PECs were collected at day 28–31 of MHV68 infection for LDAs. PECs from (Fig 1B) were disrupted before plating to detect preformed virus. Data pooled from 4 independent experiments (3 mice pooled/group). Dotted line represents Poisson distribution. (B) C57BL/6 splenocytes were collected at day 28–31 of MHV68 infection for LDAs. Splenocytes from (Fig 1C) were disrupted before plating to detect preformed virus. Data pooled from 4 independent experiments (3 mice pooled/group). Dotted line represents Poisson distribution. (C) Adult worm burden and fecundity of HP in helminth-only and coinfected mice at days 35 and 36 of HP infection, which correspond to days 28 and 29 of MHV68 infection. Data are pooled from 2 independent experiments (n = 8 mice/group, mean± standard deviation). Each dot represents an individual mouse. P-values, Unpaired t-test. * P ≤ 0.05, ** P ≤ 0.01, *** P ≤ 0.001, **** P ≤ 0.0001. (D) Littermate controls of Stat6-/- PECs from (Fig 1D) were disrupted before plating to detect preformed virus. Data are pooled from 2 independent experiments (3 mice pooled/group). Dotted line represents Poisson distribution. (TIF) [file ppat.1011691.s001.tif]

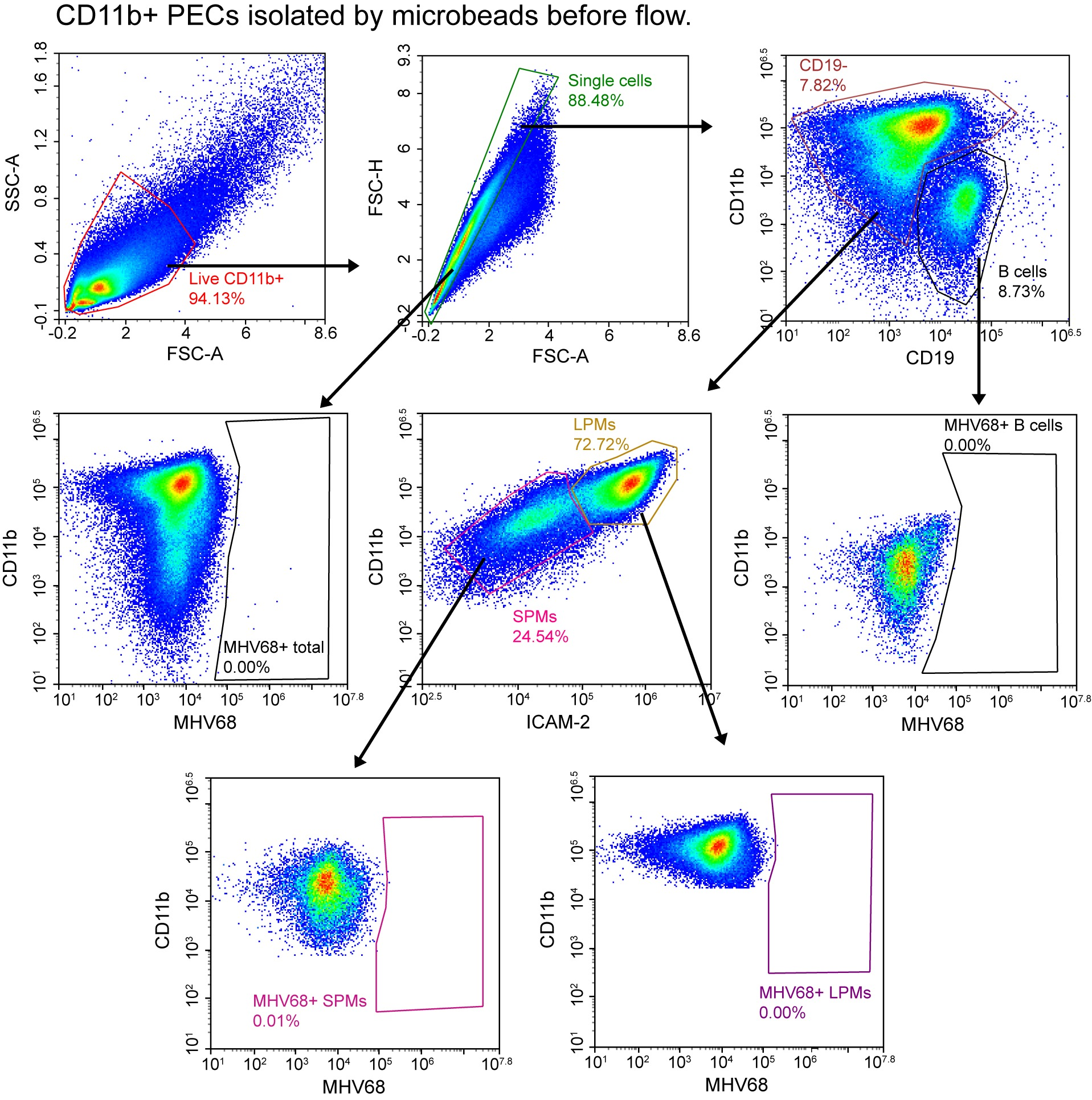

Supplement: S2 Fig — CD11b+ cells were isolated by microbeads before flow to enrich for MHV68+ cells. (TIF) [file ppat.1011691.s002.tif]

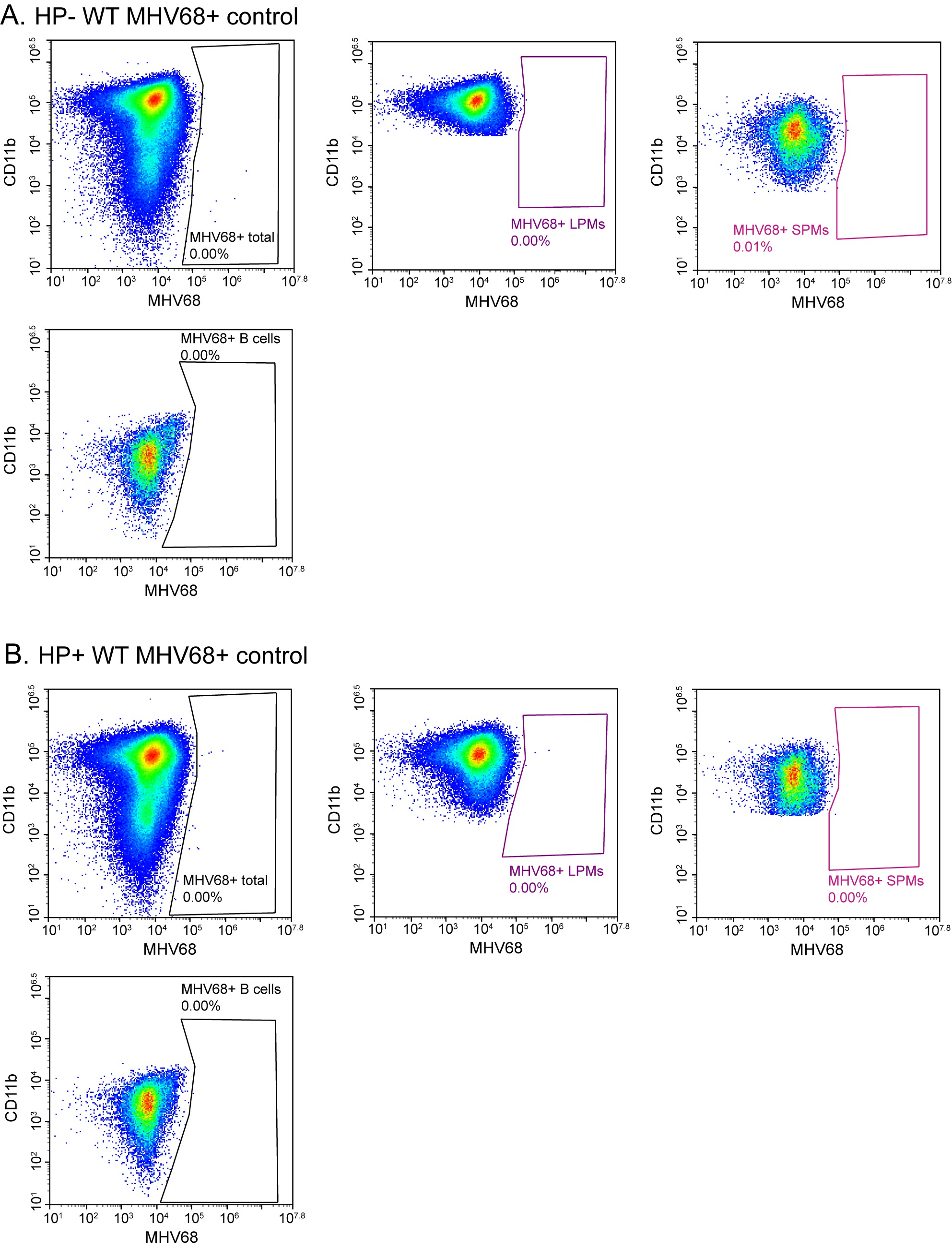

Supplement: S3 Fig — (A) Gates for βla.MHV68+ cells in virus-only mice were set on populations from WT MHV68+ mice. WT MHV68 does not produce signal with the CCF4-AM substrate. (B) Gates for βla.MHV68+ cells in HP/MHV68 coinfected mice were set on populations from HP/WT MHV68+ coinfected mice. The same strategy was used for every βla.MHV68 experiment. (TIF) [file ppat.1011691.s003.tif]

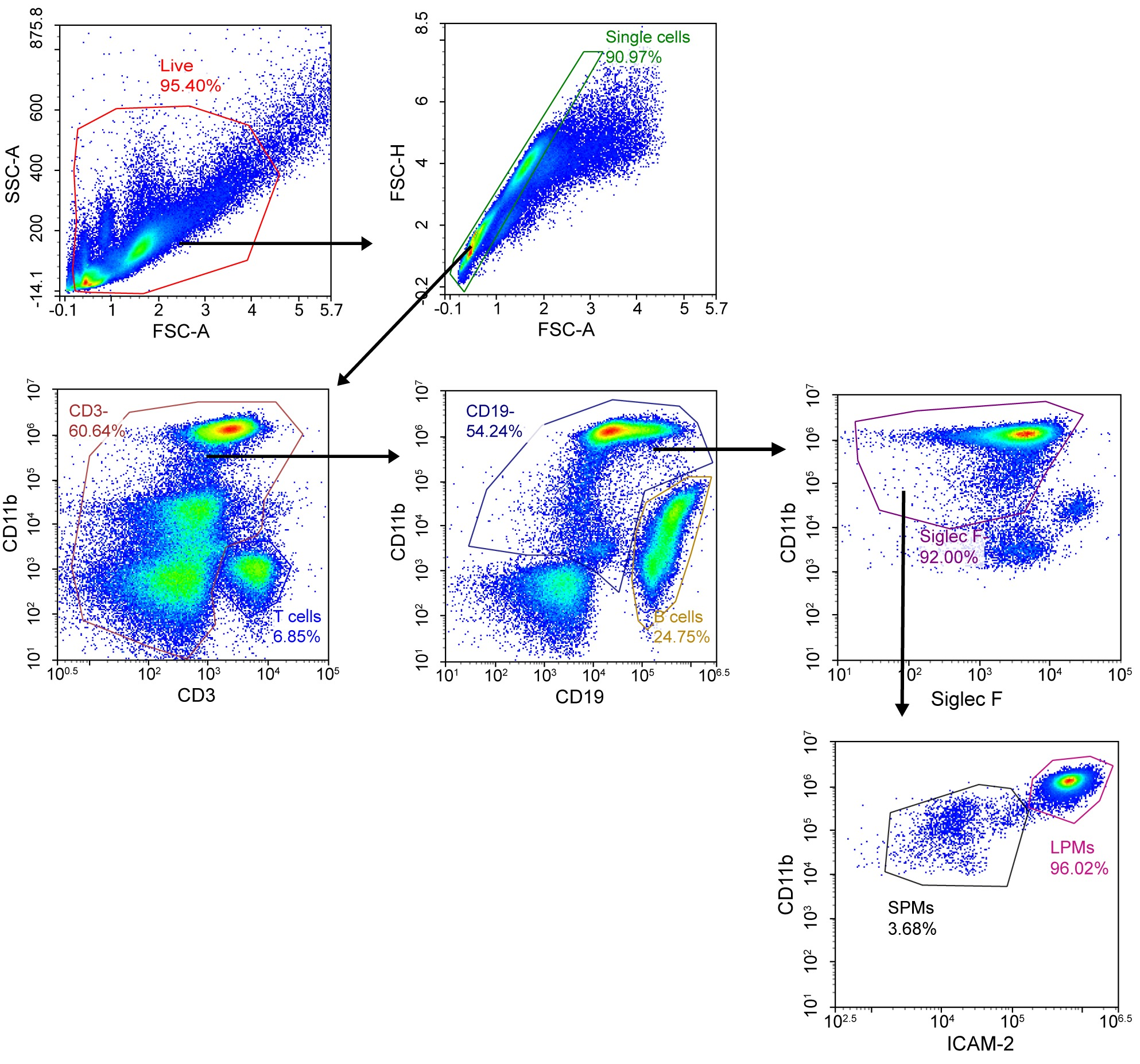

Supplement: S4 Fig — (TIF) [file ppat.1011691.s004.tif]

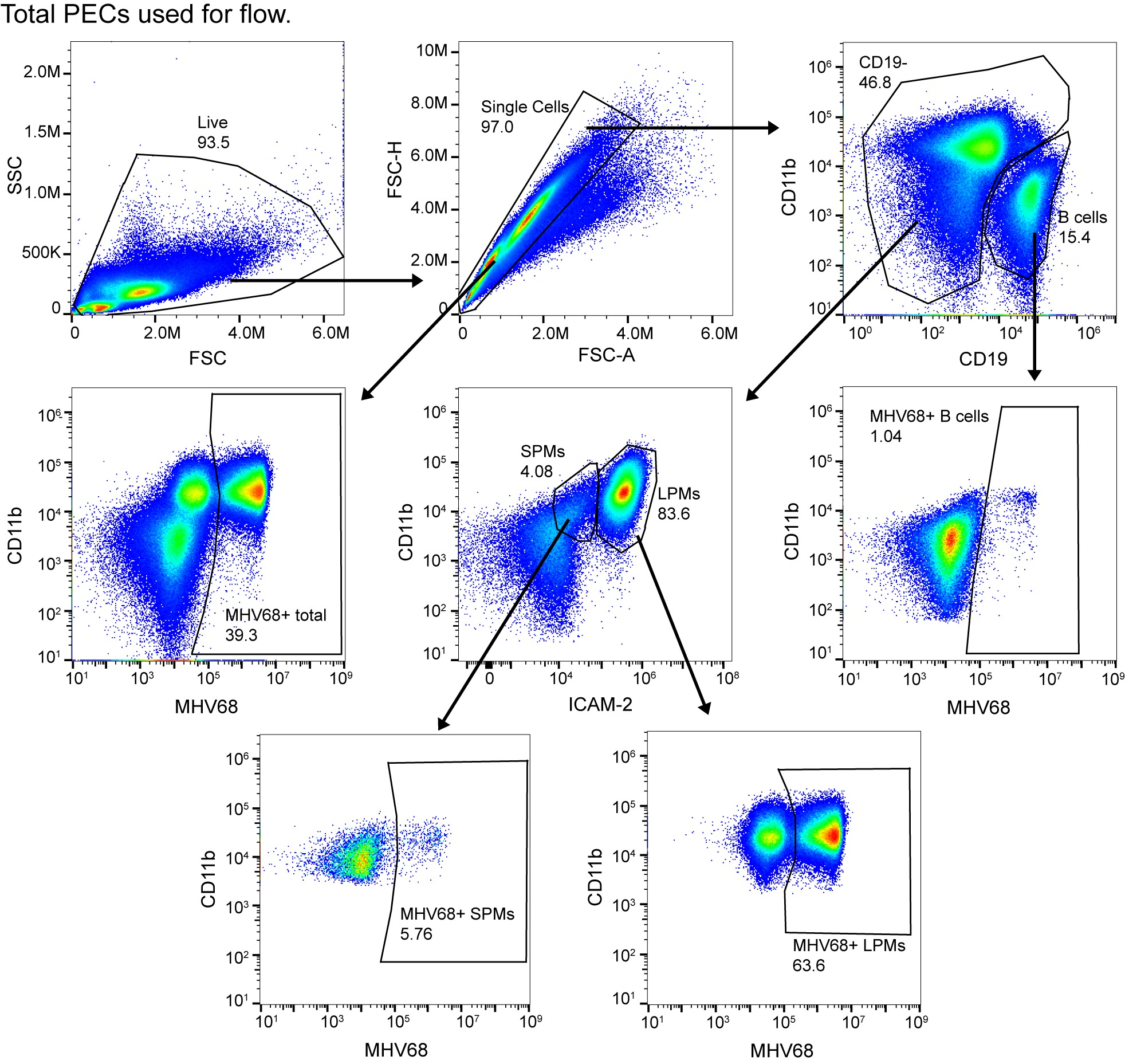

Supplement: S5 Fig — (TIF) [file ppat.1011691.s005.tif]

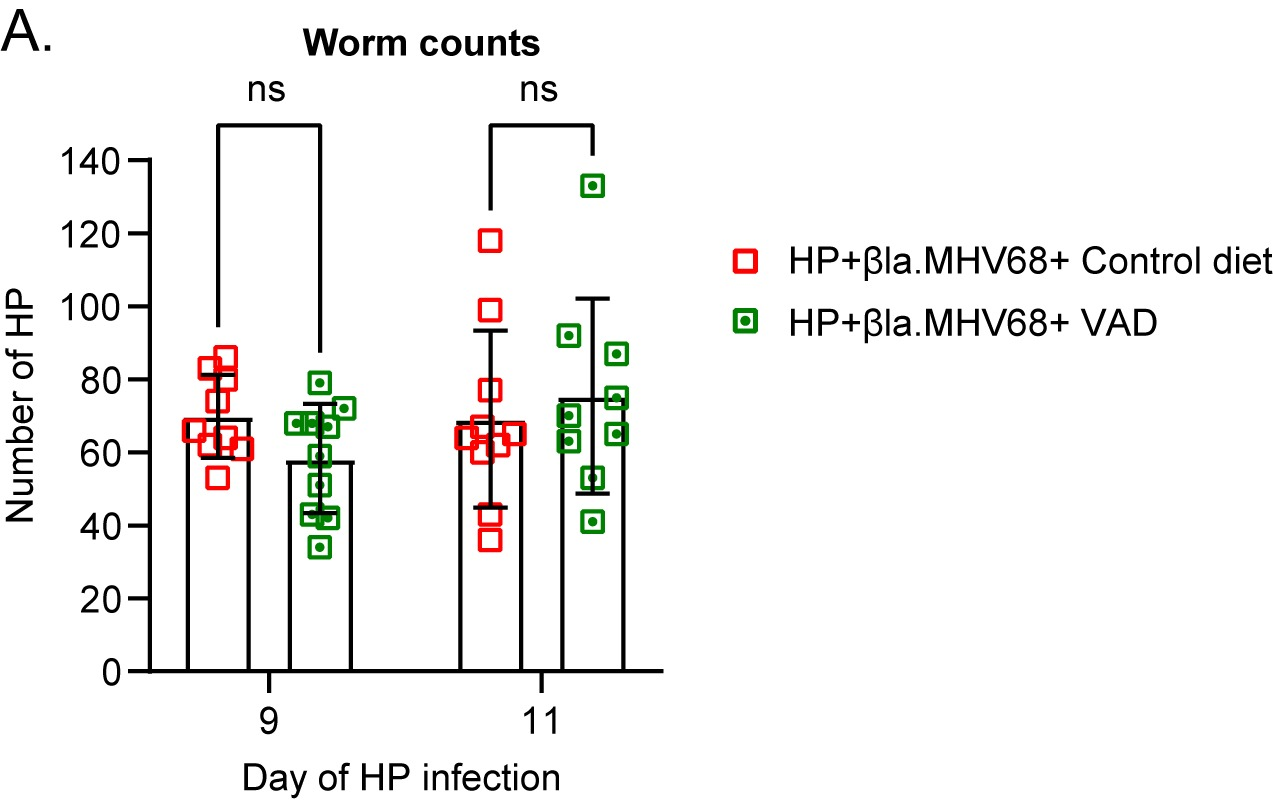

Supplement: S6 Fig — (A) Mice were raised on a vitamin A deficient diet or a control diet and infected with HP and the MHV68.ORF73β-lactamase reporter virus, as in Fig 5. Worm burden of HP at days 9 and 11 of HP infection, which correspond to days 2 and 4 of MHV68 infection. Data are pooled from 2 independent experiments (7–12 mice/group, mean± standard deviation). Each dot represents an individual mouse. P-values, 2-way ANOVA, Tukey’s multiple comparisons. * P ≤ 0.05, ** P ≤ 0.01, *** P ≤ 0.001, **** P ≤ 0.0001. (TIF) [file ppat.1011691.s006.tif]

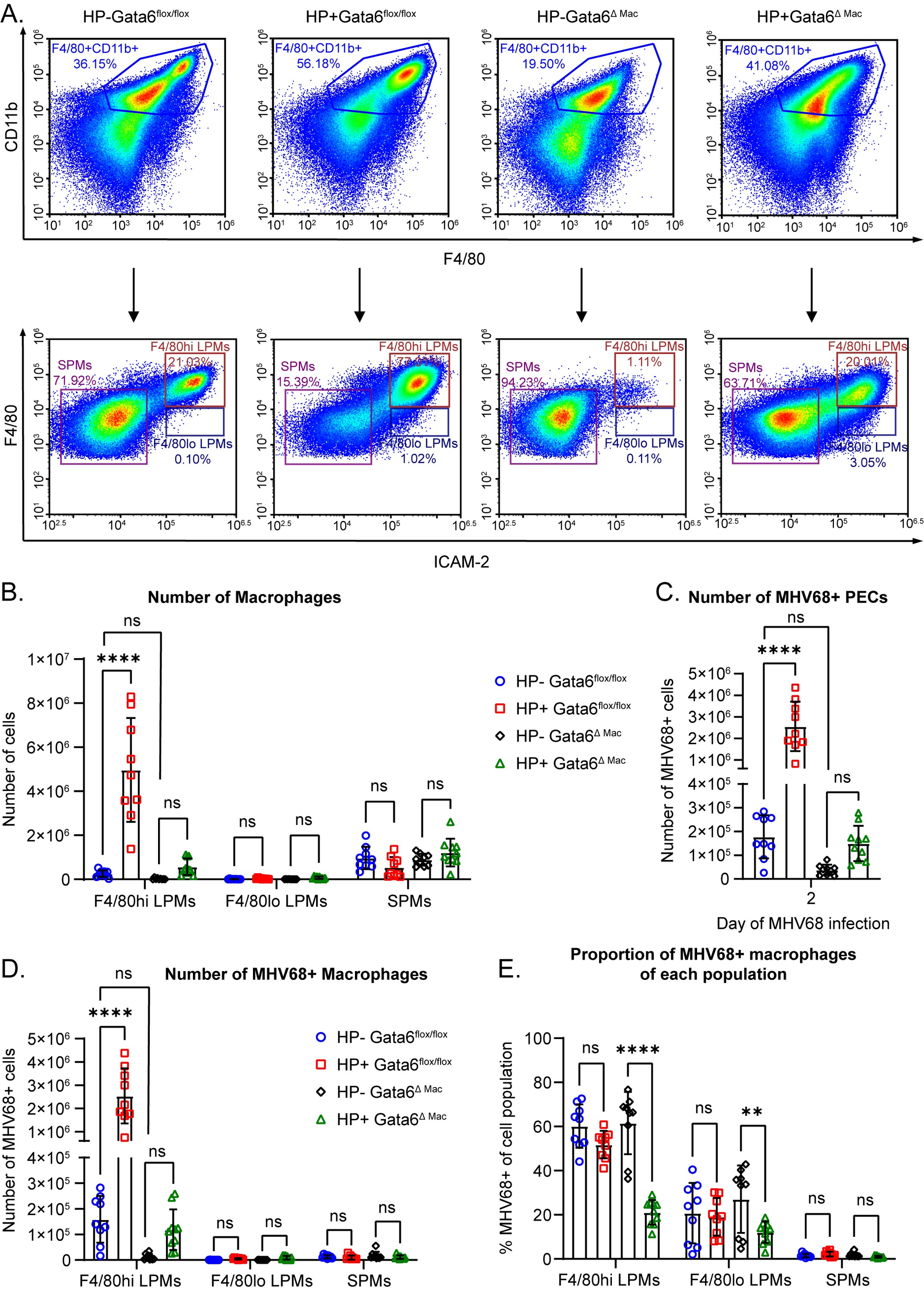

Supplement: S7 Fig — (A-E) Tamoxifen treated Gata6flox/flox and Gata6Δ Mac mice were infected with the MHV68.ORF73β-lactamase reporter virus, as in Fig 3. PECs were collected at day 2 of MHV68 infection for flow analysis. (A) Representative flow plots of macrophage gating at day 2 of MHV68 infection. (B-E) Quantification of flow cytometric analysis of MHV68-infected PECs at day 2 of MHV68 infection. Data are pooled from 2 independent experiments (n = 9-10/group, mean ± standard deviation). Each dot represents an individual mouse. (B) Number of macrophages. (C) Total number of MHV68-infected PECs. (D) Number of MHV68-infected macrophages. (E) Proportion of MHV68-infected macrophages out of the respective parent macrophage populations. P-values, 2-way ANOVA, Tukey’s multiple comparisons. * P ≤ 0.05, ** P ≤ 0.01, *** P ≤ 0.001, **** P ≤ 0.0001. (TIF) [file ppat.1011691.s007.tif]

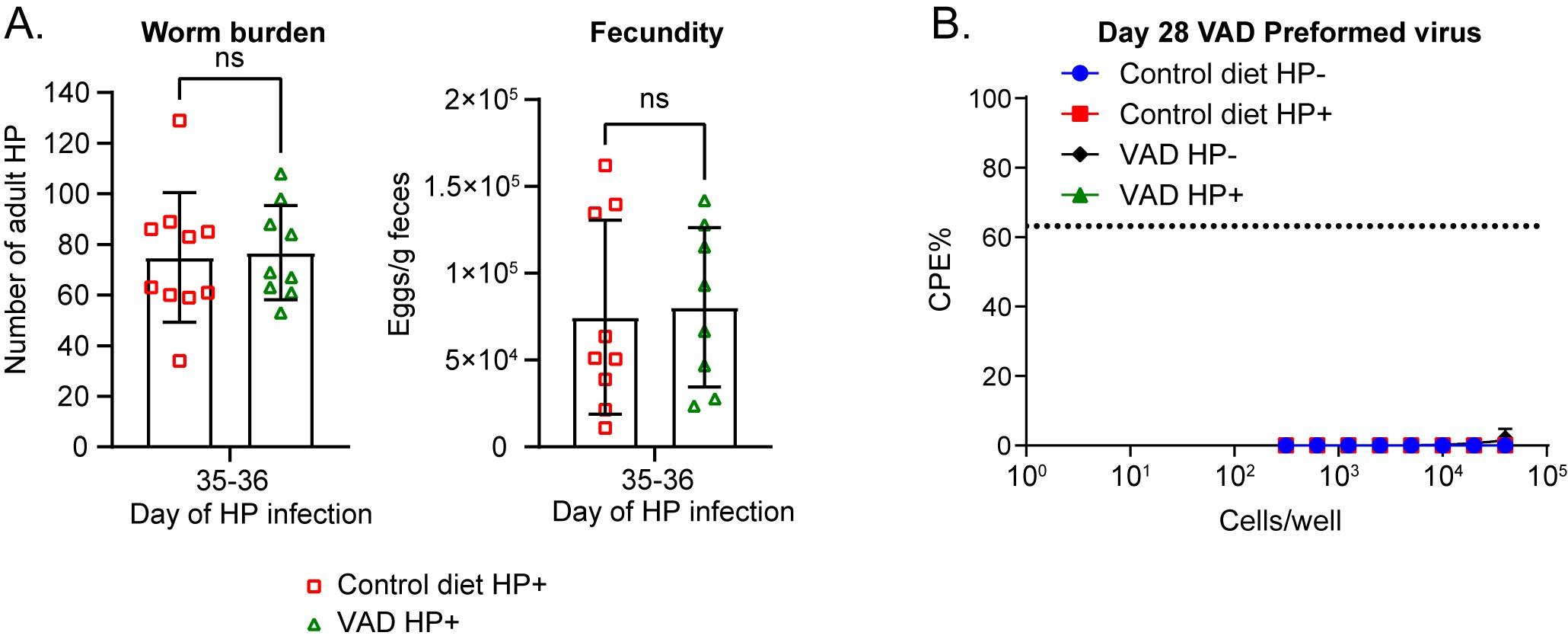

Supplement: S8 Fig — (A-B) Mice were raised on a vitamin A deficient diet or a control diet and infected with HP and the MHV68.ORF73β-lactamase reporter virus, as in Fig 7. (A) Worm burden and fecundity of HP at days 35 and 36 of HP infection, which correspond to days 28 and 29 of MHV68 infection. Data are pooled from 2 independent experiments (4–6 mice/group, mean± standard deviation). Each dot represents an individual mouse. P-values, unpaired t-test. * P ≤ 0.05, ** P ≤ 0.01, *** P ≤ 0.001, **** P ≤ 0.0001. (B) PECs from (Fig 7G) were disrupted before plating to detect preformed virus. Data are pooled from 2 independent experiments (3–5 mice pooled/group). Dotted line represents Poisson distribution. (TIF) [file ppat.1011691.s008.tif]
